# Synthesis of a Molecule with Five Different Adjacent Pnictogens

**DOI:** 10.1002/chem.202002279

**Published:** 2020-06-18

**Authors:** Christian Ritter, Florian Weigend, Carsten von Hänisch

**Affiliations:** ^1^ Department of Chemistry and Wissenschaftliches Zentrum für Materialwissenschaften (WZMW) Philipps-Universität Marburg Hans-Meerwein-Straße 4 35032 Marburg Germany; ^2^ Department of Chemistry Philipps-Universität Marburg Hans-Meerwein-Straße 4 35032 Marburg Germany

**Keywords:** bismuth, interpnictogen, main-group elements, quaternary, quinternary

## Abstract

The first molecular compound with all five pnictogens was obtained by a multi‐step reaction. Lithiation of the (bisamido)diazadiarsetidine (*t*BuNAs)_2_(*t*BuNH)_2_ in aliphatic solvents leads to the dimeric metallated species [(*t*BuNAs)_2_(*t*BuNLi)_2_]_2_ (**1_2_**). Upon reactions with AsCl_3_, SbCl_3_ and BiCl_3_ the polycyclic compounds [(*t*BuNAs)_2_(*t*BuN)_2_]PnCl (Pn=As (**2**), Sb (**3**), Bi (**4**)) can be obtained. Conversion of **2**–**4** with [*t*Bu_2_SbP(*t*Bu)Li(OEt_2_)]_2_ leads to the remarkable interpnictogens [(*t*BuNAs)_2_(*t*BuN)_2_]PnP(*t*Bu)Sb*t*Bu_2_ (Pn=As (**5**), Sb (**6**), Bi (**7**)), whereby **7** is the first example of a molecule containing all five Group 15 elements. The compound with adjacent AsNBiPSb‐chains is surprisingly stable and does not show high sensibility against light as the labile Bi−P bond might suggest.

## Graphical abstract

**Power of 5**! A molecular compound with all five pnictogen atoms and exclusively tertiary butyl substituents was obtained by a planned multi‐step synthesis (see figure).

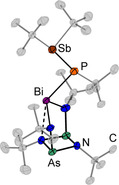

Molecular compounds that contain different Group 15 elements are of general interest due to their specific bonding situations and reactivity. Very recently the first compound with a bismuth antimony single bond could be obtained by *S. Chitnis* and co‐workers.^1^ The group of *A. Schulz* investigated in recent years the reactivity of hetrocyclic biradicaloids of the composition [E(μ‐NR)]_2_ (E=P‐Bi) and observed the activation of small molecules which exhibit single, double or triple bonds.^2^, ^3^, ^4^ Moreover, binary molecular Group 15 compound are used as precursors in semiconductor production.^5^, ^6^, ^7^ The development of new, targeted and resource‐saving methods for element‐element bond formation with Group 15 elements is therefore of general interest. Our group recently investigated the preparation of some ternary interpnictogen compounds containing adjacent Group 15 elements being substituted with *tert*‐butyl moieties solely.^8^, ^9^ The synthesis of comparable open‐chained quaternary or even quinternary molecules remains challenging and the latter one has not been realized up to now. An apparent reason might by the intrinsic instability of covalent Bi−X bonds (X=any other main group element) arising from the metallic character of bismuth and its diffuse valence orbitals. To the best of our knowledge only two quaternary interpnictogen compounds are described in the literature up to now: SbCl_2_N(Mes*)AsPMes* (Mes*=2,4,6‐tri‐*tert*‐butyl‐phenyl) by *A. Schulz* and [ClAs(*μ*‐N(=PMe_3_))_2_SbCl_4_][SbCl_6_] by *K. Dehnicke*.^10^, ^11^ Both compounds do not contain the heaviest pnictogene bismuth. Ternary interpnictogen molecules containing bismuth are often realized with phosphazene moieties like in [BiF_2_(NPEt_2_)(HNPEt_3_)]_2_, (tms)N[P(NMe_2_)_2_N]_2_Bi(OAc) (tms=trimethylsilyl) or C[Ph_2_P=N(dipp)]_2_BiCl (dipp=2,6‐diisopropyl‐phenyl).^12^, ^13^, ^14^ Furthermore, the compound O[SiMe_2_N(R)]_2_BiP(cy)_2_ (cy=cyclohexyl; R=dipp, *t*Bu) should be mentioned as it contains a covalent Bi−N and Bi−P bonds.^15^ Also known is {O[SiMe_2_N(R)]_2_Bi}_2_P_4_, which contains an activated P_4_ moiety with a butterfly‐like shape between two Bi atoms.^15^ Pentavalent bismuth species like Ph_3_Bi(N=PPh_3_)_2_ remain exceptional within this class of compounds.^16^

There are a lot of diazadiphosphetidines described in the literature with a wide range of different substitution patterns^17^, ^18^, ^19^, ^20^, ^21^ but only a few with antimony or bismuth moieties like in (PNR^1^)_2_(NR^1^)_2_PnR^2^ (Pn=Sb: R^1^=*t*Bu, Ph; R^2^=Cl, N_3_, OPh, N(tms)_2_; Pn=Bi R^1^=*t*Bu; R^2^=Cl).^22^ Worth mentioning are also some mixed diazapnictitidines by *A. Schulz* et al. like for example (*μ*‐NTer)_2_PSbCl_2_ (Ter=2,6,‐bis(2,4,6‐trimethyl‐phenyl)phenyl) which can be reduced to the biradicaloid [(*μ*‐NTer)_2_PSb] or to the eight‐membered heterocycle of the type Sb_2_[*μ*‐(NTer)_2_P]_2_.^23^ Diazadistibetidines were first synthesized in the 1970s by *Scherer* and *Kuhn* and since then several derivatives have been mentioned in the literature.^24^ Besides some halide substituted compounds like [X‐Sb(*μ*‐NR)]_2_ (X=halide; R=Mes*, *t*Bu), there are also some further functionalized ones like [R^1^Sb(*μ*‐NR^2^)]_2_ (R^1^=*t*Bu; R^2^=N_3_, OPh, O*t*Bu, OMe, *t*Bu, Me, PSiMe_3_. R^1^=NH*t*Bu, NHDipp, NHDmp; R^2^=*t*Bu (Dmp=2.6‐dimethyl‐phenyl)).^25^, ^26^, ^27^, ^28^ Diazadibismetidines like [Bi_2_(N*t*Bu)_4_Li_2_], [PhBi(*μ*‐N*t*Bu)]_2_ or [DippNHBi(*μ*‐NDipp)]_2_ are known as well but only a little work has been done in this field up to now.^29^, ^30^, ^31^ In the class of diazadiarsetidines, there are only a few compounds known containing Bi or Sb: (AsN*t*Bu)_2_(N*t*Bu)_2_BiCl and (AsNR)_2_(NR)_2_SbCl (R=*t*Bu, amyl) are the only examples, but for the one with bismuth there are no additional information or analytical data given.^32^, ^33^ This lack of knowledge lead us to the idea of focusing more intensively on this class of compounds and to use them as starting point for the first quinternary species of Group 15 elements (see Scheme 1 and Supporting Information for details).

**Scheme 1:**
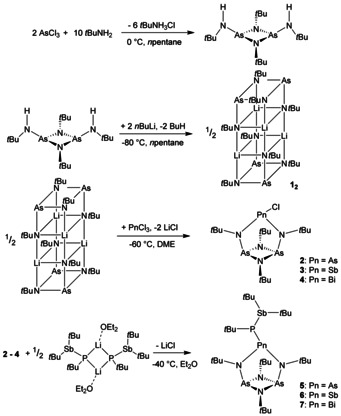
Reaction Scheme for the preparation of the (bisamido)diazadiarsetidine,^32^ its lithiation to **1_2_**, as well as the further conversion with the pnictogen trichlorides to **2**–**4** and finally the reaction with the lithiated di‐*tert‐*butyl‐stibino‐*tert‐*butyl‐phosphane to **5**–**7**.

The first step preparing quaternary and quinternary interpnictogen compounds is the lithiation of the (bisamido)diazaarsetidine (*t*BuNAs)_2_(*t*BuNH)_2_, which was already done by *Veith* and co‐workers in 1994, but not analyzed sufficiently.^32^ [(*t*BuNAs)_2_(*t*BuNLi)_2_] (**1**) can be prepared in good yields (79 %) by adding two equivalents of a *n*BuLi solution to (*t*BuNAs)_2_(*t*BuNH)_2_ in *n‐*pentane.

Crystallization of **1** from *n‐*pentane or DME (DME=1,2‐dimethoxyethane) yields different molecular structures: When using *n‐*pentane, **1** crystallizes in the monoclinic space group *C*2/*c* as a dimer ([(*t*BuNAs)_2_(*t*BuNLi)_2_]_2_ (**1_2_**)). The molecule consists of three stacked distorted cubes so that each lithium ion is coordinated by four nitrogen atoms (Figure 1 left). Furthermore, agostic interactions of the methyl groups of the *tert‐*butyl moieties to Li can be observed leading to a distorted trigonal bipyramidal coordination of each lithium atom. A similar structure motive has been observed within [(cyNAs)_2_(cyNLi)_2_]_2_, but the segments deviate more from ideal cube‐like shapes leading to a distorted tetrahedral conformation of N and Li.^34^

**Figure 1:**
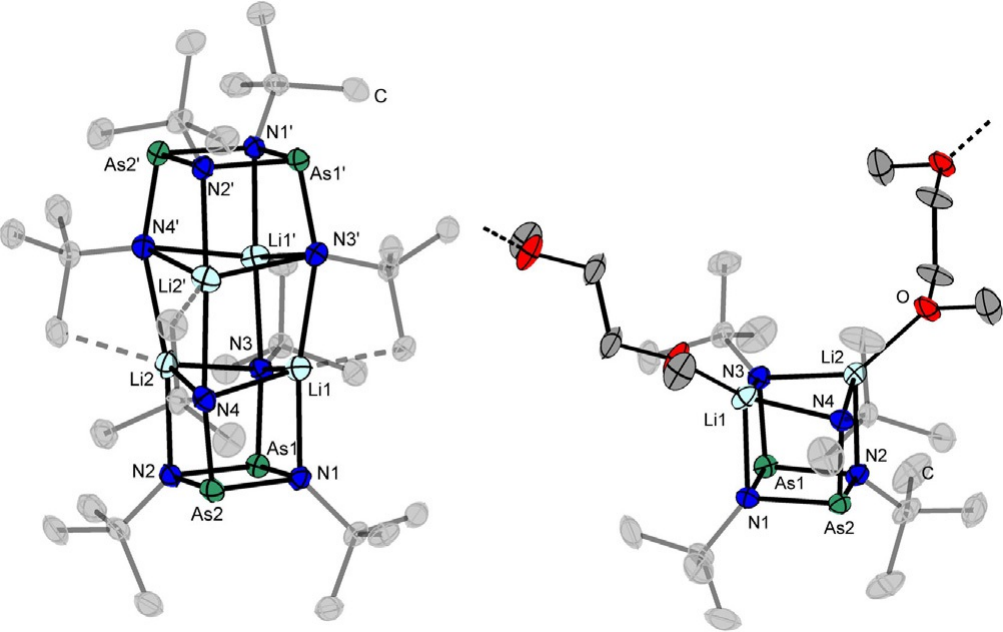
Molecular structures of **1_2_** (left) and **1⋅dme** (right) in the solid state. Agostic interactions and coordination to the next formula unit are depicted as dotted lines. Carbon atoms of the *tert*‐butyl groups are shaded and hydrogen atoms are omitted for clarity. Displacement ellipsoids represent a 50 % probability level at 100 K. Selected bond lengths [Å] and angles [°] of **1**: As1/2−N1/2 1.913(2)–1.921(2), As1/2−N3/4 1.822(2)–1.830(3), Li1/2−N1/2 2.111(6)–2.122(6), Li1/2−N3/4 2.187(5)–2.268(8), N1‐As1/2‐N2 83.5(1)–83.9(1), N1/2‐Li1/2‐N3’/4’ 169.3(3)–169.6(3), N3‐Li1/2‐N4 104.4(2)–105.2(2); **1⋅dme**: As1/2‐N1/2 1.914(5)–1.919(5), As1/2‐N3/4 1.805(5)–1.808(5), Li1/2‐N1/2 2.11(1)–2.11(1), Li1/2‐N3/4 2.07(1)–2.08(1), Li‐O 1.97(3)–2.07(3), N1‐As1/2‐N2 82.7(2)–82.8(2), N1/2‐Li‐O 123.6(9)–130.1(8).

When crystallizing from DME, the structure consists of infinite chains of monomeric units of **1** being bridged with one molecule of DME on each Li atom (**1⋅dme**, Figure 1 right). It crystallizes in the monoclinic space group *P*2_1_/*n* with four formula units per unit cell.

When adding an equimolar solution of PnCl_3_ (Pn=As, Sb, Bi) to **1** dissolved in DME at −60 °C the tricyclic compounds **2**–**4** can be obtained in good to excellent yields (62–95 %). **3** and **4** have already been prepared in the past, but for **4** no analytical data were provided.^32^, ^33^ NMR spectroscopy in C_6_D_6_ of the compounds **2**–**4** was conducted at room temperature and for the ^1^H nucleus (300 MHz) the results unexpectedly differ (see Supporting Information for details). For **2** the spectrum exhibits two singlets: One located at 1.24 ppm with an integral of 9 and the other one at 1.46 ppm with an integral of 27 hydrogen atoms. The spectrum for **3** shows three singlets at 1.10, 1.31 and 1.64 ppm with integrals of 9, 9 and 18 protons and for **4** two singlets can be observed at 1.18 and 1.51 ppm with integrals of 18 protons each. To resolve these discrepancies, temperature dependent ^1^H NMR spectra for **2**–**4** were conducted in [D_8_]toluene (500 MHz) in a range from 190 K to 350 K (see Figures S18–S20). Therein it can be seen that at 190 K for all compounds (**2**–**4**) the signals for the *tert‐*butyl moieties located at the (AsN)_2_ ring split into two signals with an integral of 9 protons each. Coalescence can be observed for **4** at a value of about 250 K. For **2** and **3** the same trend is observable, whereby an exact temperature of coalescence cannot be determined. Due to increasing broadening of the signals of **2** it can be assumed that coalescence probably occurs at around 360 K. The counterintuitive pattern of the signals of **2** at room temperature can be accounted to a crossing of the signals randomly leading to a sharp singlet with the integral of 27 protons and a second signal with an integral of 9. The coalescence in **2** can be explain by swinging of the As‐Cl entity from one side to the other. **3** and **4** show additional coordination sites of the Sb/Bi atom in the solid‐state structure (see below) and most likely a pyramidal inversion takes place by changing the coordinating nitrogen atom. Such an inversion was also described within the analogous (bisamido)diazadiphosphetidine by *L*. *Stahl* and co‐workers.^22^ Calculation of the activation energy for the inversion of **2**–**4** using the line shape analysis yields an activation energy of 59(2) kJ mol^−1^ for **2**, 62(3) kJ mol^−1^ for **3** and 47(3) kJ mol^−1^ for **4**. The reaction of **1** with PCl_3_ was also carried out a couple of times, but as can be seen in the solid‐state structure (Figure S1) a reorganization of the bicyclus takes place and a mixed AsN_2_P ring is formed.

Suitable single crystals of compound **2** can be obtained from a solution in toluene at −30 °C as colourless blocks. It crystallizes in the monoclinic space group *P*2_1_/*m* with two formula units per unit cell (Figure 2). The structure is isotype to the solid state structures of the analogous diazadipnictetidines (*t*BuNP)_2_(*t*BuN)_2_PnCl (Pn=P, As).^19^

**Figure 2:**
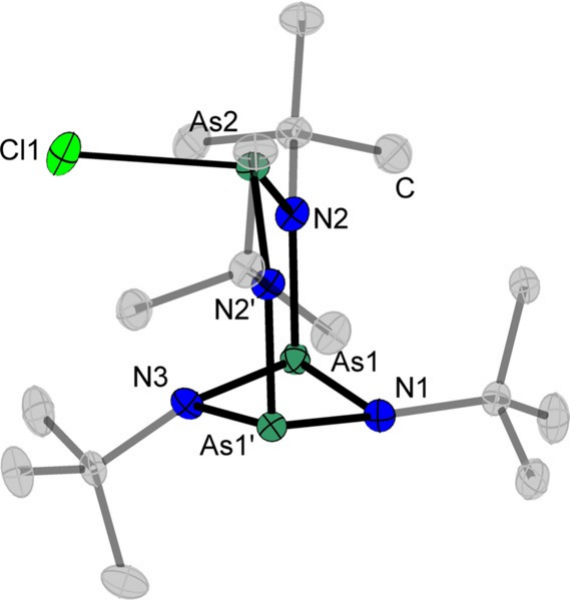
Molecular structures of **2** in the solid state. Carbon atoms of the *tert*‐butyl groups are shaded and hydrogen atoms are omitted for clarity. Displacement ellipsoids represent a 50 % probability level at 100 K. Selected bond lengths [Å] and angles [°]: N1/3−As1 1.848(2)–1.854(2), As1−N2 1.887(2), N2−As2 1.823(2), As2−Cl1 2.3730(11), N1‐As1‐N3 81.34(10), As1‐N1/3‐As1’ 96.8(1)–97.3(1), As1‐N2‐As2 124.83(11), N2‐As2‐N2 103.8(1), N2‐As2‐Cl1 102.17(7).

Single crystals of compound **4** can also be obtained from toluene at −30 °C as yellow blocks. It crystallizes with one formula unit per unit cell in the triclinic space group *P* $\bar{1}$ containing additionally half a molecule of toluene. In the solid state, compound **4** is dimeric due to an additional secondary bonding from a chlorine atom to the bismuth atom of the next molecule. Due to a further coordination of the Bi atom by N2 the coordination number of Bi is five (Figure 3).

**Figure 3:**
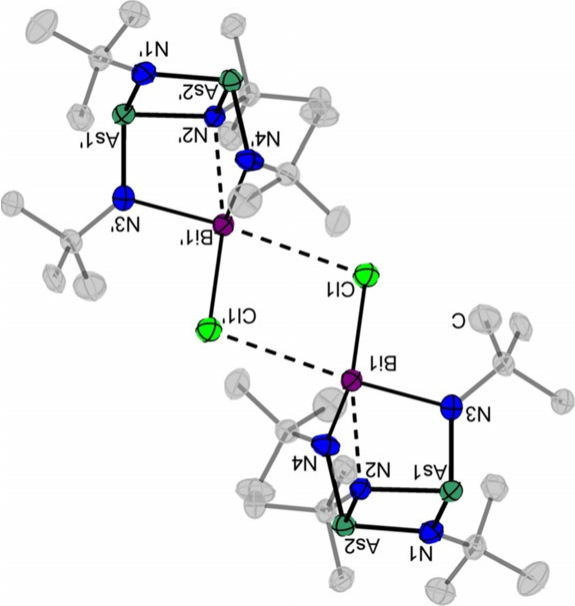
Molecular structures of **4** in the solid state. Secondary bonding interactions are depicted as dotted lines. Carbon atoms of the *tert*‐butyl groups are shaded and hydrogen atoms as well as the co‐crystalline molecule of toluene are omitted for clarity. Displacement ellipsoids represent a 50 % probability level at 100 K. Selected bond lengths [Å] and angles [°]: As1/2−N1 1.852(8)–1.856(8), As1/2−N2 1.913(8)–1.928(8), As1/2−N3/4 1.827(9)–1.839(9), N3/4−Bi1 2.182(8)–2.204(8), N2−Bi1 2.418(7), Bi1−Cl1 2.713(2), Bi1−Cl1’ 3.500(2), N1‐As1/2‐N2 79.4(3)–79.7(3), As1‐N2‐As2 97.9(3), As1‐N1‐As2 102.7(4), N3‐Bi1‐N4 98.4(3), Bi1‐Cl1‐Bi1’ 86.23(6).

When using the compounds **2**–**4** as educts for further conversion with the lithiated di‐*tert‐*butyl‐stibino‐*tert‐*butyl‐phosphane, the compounds [(*t*BuNAs)_2_(*t*BuN)_2_]PnP(*t*Bu)Sb*t*Bu_2_ (Pn=As, Sb, Bi) **5**–**7** can be obtained in moderate to good yields (Scheme 1).^8^ **5** and **6** represent novel quaternary and **7** the first quinternary interpnictogen molecule ever described. For the preparation it is of crucial importance to work with exact stoichiometries due to a hindered crystallization when byproducts or excessive reactants are present. However, when trying to synthesize **5**, the reaction is simply too unselective to obtain a clean product. The ratios of the different compounds can be affected by varying solvents and temperature, but the amount of different compounds being formed is too high and a fractional crystallization was not successful. It was once possible to obtain single crystals of the compound **5** to perform single crystal X‐ray diffraction, but due to bad crystal quality the data were of poor quality (Figure S2). One side product was identified as the diarsane [{(*t*BuNAs)_2_(*t*BuN)_2_}As]_2_ formed by reduction of **2**. In the ^31^P{^1^H} NMR spectra of **5**–**7** the arsenic compound **5** shows a significantly more low‐field‐shifted signal at 61.7 ppm compared to **6** or **7** with 22.5 and 37.6 ppm, respectively.

Compounds **5** and **6** crystallize isomorph from *n‐*pentane at −30 °C in the monoclinic space group *Cc* with two independent molecules in the asymmetric unit (Figure 4). In compound **6** the bonds from the bridging Sb to the phosphorus atom are arranged nearly parallel to the (AsN)_2_ ring and have lengths of 2.582(2) Å and 2.590(2) Å. They are slightly longer than the other Sb−P bonds in the molecule with 2.541(1) Å or in other known compounds with Sb−P single bonds.^8^, ^9^, ^35^ The Sb‐P‐Sb angles in **6** are with 93.83(5)–94.78(5)° significantly smaller than in [*t*BuPh_2_SiP{SbClCH(tms)_2_}_2_] (99.39(6)°) or in *t*BuP[Sb(*t*Bu)_2_]_2_ (109.22(2)°).^8^, ^36^

**Figure 4:**
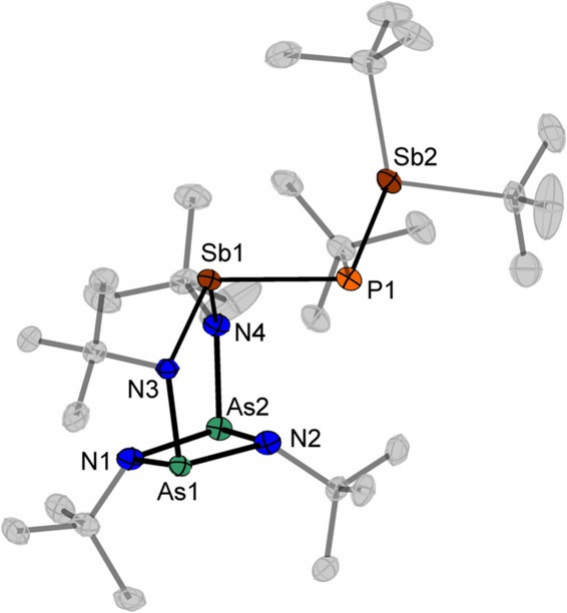
Molecular structure of **6** in the solid state. Carbon atoms of the *tert*‐butyl groups are shaded and hydrogen atoms are omitted for clarity. Displacement ellipsoids represent a 50 % probability level at 100 K. Selected bond lengths [Å] and angles [°]: As1/2−N1/2 1.867(4)–1.910(5), As1/2−N3/4 1.841(5)–1.867(4), N3/4−Sb1 2.078(5)–2.091(5), Sb1−P1 2.582(2)–2.590(2), Sb2−P1 2.541(2), N1‐As1/2‐N2 84.4(2)–84.9(2), As1‐N1/2‐As2 94.1(2)–96.5(2), N3‐Sb1‐N4 103.3(2)‐104.2(2), Sb1‐P1‐Sb2 93.83(5)–94.78(5).

Compound **7** crystallizes from *n‐*pentane at −30 °C in the triclinic space group *P* $\bar{1}$ with two formula units per unit cell (Figure 5). In contrast to the solid‐state structures of **5** and **6** an additional coordination from N2 to the central metal atom (bismuth) is observed. This leads to a more central position of the P atom above the (AsN)_2_ ring. With 2.833(7) Å the N2−Bi1 interaction is not as distinct as in **4** or in other molecules like in Bi(CN)_3_(thf)_2_ (2.733(7) Å), in Bi(SCN)Ph_2_⋅0.5 CHCl_3_ (2.52(5)–2.53(6) Å) or in Bi(N_3_)_3_ (2.578(7)–2.685(6) Å), although it should be mentioned that the coordination number is different in the various compounds.^37^, ^38^, ^39^ However, the N2–Bi1 distance in **7** is significantly shorter than the sum of the VdW radii (3.62 Å).^40^ The Bi−P bond length has a value of 2.751(3) Å and thus is longer than in (*t*Bu)_2_BiP(*t*Bu)Sb(*t*Bu)_2_ (2.624(3) Å), SIMesPBi(*t*Bu)_2_ (2.603(2) Å) or [*t*BuPh_2_SiP{BiClCH(tms)_2_}_2_] (2.626(2)–2.651(2) Å).^9^, ^35^, ^36^ The Sb−P bond length (2.550(2) Å) is similar to that in **6** (2.541(2) Å), SIMesPSb(*t*Bu)_2_ (2.5031(5) Å) or in [*t*BuPh_2_SiP{SbClCH(tms)_2_}_2_] (2.545(2)–2.553(2) Å).^9^, ^35^, ^36^ The Bi‐P‐Sb angle in **7** (102.20(8)°) is clearly larger than the Sb‐P‐Sb angle in **6** (93.83(5)–94.78(5)°), but smaller than that in (*t*Bu)_2_BiP(*t*Bu)Sb(*t*Bu)_2_ (107.91(11)°) which can be accounted to the higher sterical demand of the (bisamido)diazadiarsetidines substitution in **7**.^9^

**Figure 5:**
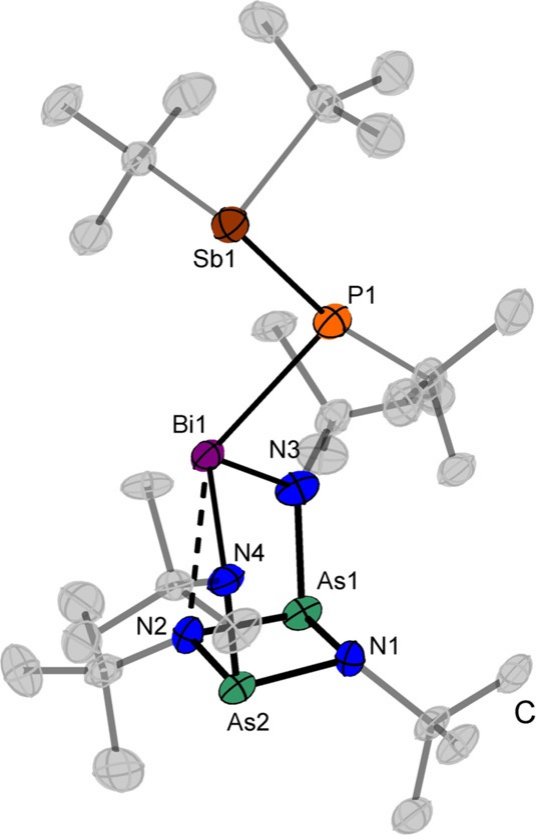
Molecular structure of **7** in the solid state. Secondary bonding interactions are depicted as dotted lines. Carbon atoms of the *tert*‐butyl groups are shaded and hydrogen atoms are omitted for clarity. Displacement ellipsoids represent a 50 % probability level at 100 K. Selected bond lengths [Å] and angles [°]: As1/2−N1 1.863,(6)–1.871(6), As1/2−N2 1.879(6)–1.886(6), N3/4−Bi1 2.212(6)–2.241(7), Bi1−N2 2.833(7), Bi1−P1 2.750(2), P1‐Sb1 2.549(2), As1‐N1‐As2 99.7(3), As1s‐N2‐As2 98.6(3), N1‐As1/2‐N2 80.5(3)–80.9(3), N3‐Bi1‐N4 104.1(2), Bi1‐P1‐Sb1 102.21(7).

For the bismuth containing compounds **4** and **7** it might be worth mentioning that they are remarkably stable against light and room temperature. Especially in the case of **4**, the compound can be stored infinitely at room temperature under irradiation of light and even when heating there are no signs of decomposition observable (e.g. formation of bismuth black). The reason for this might be the stabilizing effect of the chelating bisamido substitution. Also, **7** can be easily handled at room temperature, it decomposes only very slowly within weeks or months when stored under the influence of light but does not show any sensitivity to room temperature when stored in the dark. The reason for the relatively high stability of this compound might be the alternation of high and low electronegativities in this interpnictogen chain. In particular, the bismuth atom is protected by the chelating effect of the two nitrogen substituents as well as the additional coordination of a nitrogen atom from the four membered (AsN)_2_ ring and shows only one additional bond to a phosphorus atom.

In contrast to the compounds **1**–**6** which are colourless or yellow, **7** has an intensive red colour. In the UV/Vis spectrum of **7**, a maximum appears in the UV region at 352.5 nm with molar extinction coefficients of 7.2×10^5^ l mol^−1^ m^−1^ (Figure S4). Furthermore there is a strong band in the visible region located at 441.5 nm with a molar extinction coefficient of 2.9×10^5^ l mol^−1^ m^−1^, explaining the intensive red colour of this compound. For comparison we also measured the UV/Vis Spectrum of **6** which only shows minor absorbance in the visible region. For the elucidation of these differences we calculated^41^ the electronic excitation energies for both compounds with time‐dependent density functional theory using the PBE0 functional^42^ for optimized structure parameters. We employed the quasi‐relativistic (one‐electron) exact two‐component method, X2C,^43^, ^44^ with triple zeta bases^45^ and integration grids^46^ optimized for this purpose. The experimental results are well reproduced, as shown in Figure S5, apart from a consequent blueshift by ca. 30 nm. In particular, like in the measured spectra, in **7** the first excitation peak (at 411 nm, marked in red in Figure S5) is well separated from the others, while in **6** it is not (first peak at 348 nm). In both compounds, the first excitation has almost pure HOMO–LUMO character, the HOMO in both cases is localized dominantly at the P atom, the LUMO shows large contributions from Bi1/Sb1. The difference in the excitation energies of ca. 0.5 eV is in line with the difference of the HOMO–LUMO gaps (ca. 0.6 eV), which mainly arises from different LUMO energies (**7**: −1.11 eV, **6**: −0.622 eV). Responsible for these differences are both the change of the pnictogene and the slightly different molecular structure. In order to show this, we optimized two hypothetical structurers, **6Bi** with Bi at the central position starting from the structure parameters of **6**, and **7Sb** with Sb at the central position starting from the structure parameters of **7**. For both structures, which are about 5 kJ mol^−1^ higher in energy than the corresponding original structures **6** and **7**, no low‐energy peak is found, but the first excitations are close to that of compound **6** (350 nm for **6Bi** and 366 nm for **7Sb**).

Based on the lithiated (bisamido)diazadiarsetidine (compound **1**), we prepared the tricyclic interpnictogen compounds [(*t*BuNAs)_2_(*t*BuN)_2_]PnCl (Pn=As (**2**), Sb (**3**), Bi (**4**)).^32^ The synthesis of the analogue phosphorus compound (Pn=P) failed due to an uncontrollable reorganisation that always leads to a mixture of products. By conversion of **2**–**4** with the lithiated stibinophosphine [(*t*Bu)_2_SbP(*t*Bu)Li(OEt_2_)]_2_ 8 we prepared two novel quaternary interpnictogen compounds (**5** and **6**) and the first example of an molecule containing all five reasonable Group 15 elements in a row [(*t*BuNAs)_2_(*t*BuN)_2_]BiP(*t*Bu)Sb*t*Bu_2_ (**7**). Focus of current research is on the use of interpnicogen compounds for semiconductor preparation and the synthesis of a quinternary Group 15 molecular compound in the order of the periodic table.

## Experimental Section

### General

See the Supporting Information.

### CCDC

Deposition Number(s) 1983180 (**1_2_**), 1983179 (**1⋅dme**), 1983177 (**2**), 1983182 (**4**), 1983178 (**5**), 1983184 (**6**) and 1983183 (**7**) contain(s) the supplementary crystallographic data for this paper. These data are provided free of charge by the joint Cambridge Crystallographic Data Centre and Fachinformationszentrum Karlsruhe Access Structures service www.ccdc.cam.ac.uk/structures.

## Conflict of interest

The authors declare no conflict of interest.

## Supporting information

As a service to our authors and readers, this journal provides supporting information supplied by the authors. Such materials are peer reviewed and may be re‐organized for online delivery, but are not copy‐edited or typeset. Technical support issues arising from supporting information (other than missing files) should be addressed to the authors.

Supplementary
