# Supplementary material for: Synthesis of a Molecule with Five Different Adjacent Pnictogens
Source: Chemistry. 2020 Jun 18;26(39):8536–40. doi: 10.1002/chem.202002279 (PMC7383748; doi:10.1002/chem.202002279)
Supplement: Supplementary file 1 — Supplementary [file CHEM-26-8536-s001.pdf]

Chemistry–A European Journal

Supporting Information

Synthesis of a Molecule with Five Different Adjacent Pnictogens

Christian Ritter,[a] Florian Weigend,[b] and Carsten von Hänisch*[a]

Content

1 Solid State Structures S2

2 Experimental S3

3 X-Ray Structure Analysis S7

4 UV-Vis Spectra, measured and calculated S9

5 Mass-Spectra S11

6 NMR Spectra S15

7 References S26

1 Solid State Structures

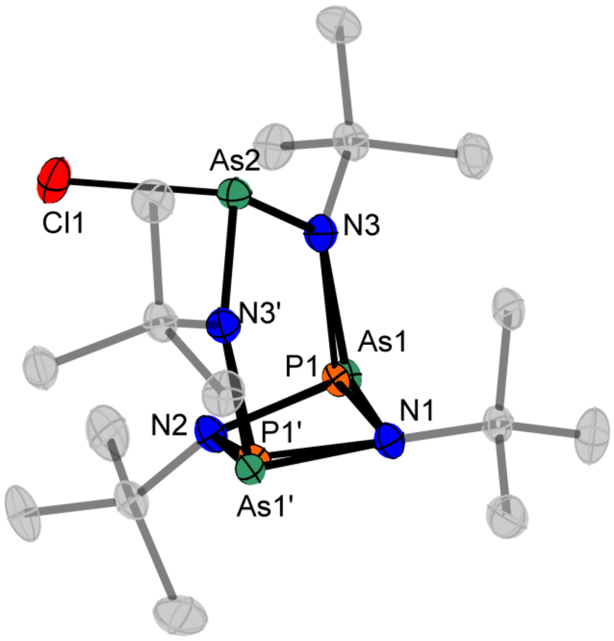

Figure S1. Molecular structure of $[As_{1.09}P_{0.91}(N\textit{t}Bu)_2](\textit{t}BuN)_2AsCl$ in the solid state. Ratio of P/As occupancy ≈ 0.45/0.55. Carbon atoms are shaded and hydrogen atoms are omitted for clarity. Displacement ellipsoids represent a 50 % probability level at 100 K.

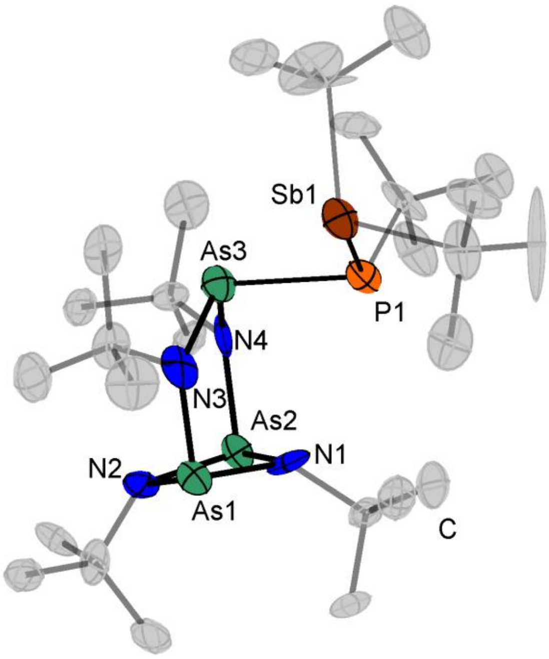

Figure S2. Molecular Structure of **5** in the solid state Carbon atoms are shaded and hydrogen atoms are omitted for clarity. Displacement ellipsoids represent a 50 % probability level at 100 K.

2 Experimental

All manipulations were performed under an inert argon atmosphere using standard Schlenk techniques. The solvents were dried by standard procedures and freshly distilled before being used.[1] The storage and use of moisture or air sensitive substances are carried out under an inert argon atmosphere in a glovebox. (tBu$_2$SbP[Li·OEt$_2$]tBu)$_2$[2] (tBuNAs)$_2$(tBuNH)$_2$[3] were prepared according to literature methods. $AsCl_3$ and $BiCl_3$ were purchased from Sigma Aldrich, nBuLi solution from Acros Organics and $SbCl_3$ from Alfa Aesar. $SbCl_3$ and $BiCl_3$ were purified by sublimation prior to use. NMR spectra were recorded at 300 K on a Bruker AV II 300, AV III HD 300 and AV III 500.The coupling constants J were reported in Hertz (Hz) and the chemical shift (δ) is given in ppm relative to the standard (^{31}P: H_3PO_4; ^{1}H, ^{13}C: SiMe$_4$). IR spectra were recorded on a Bruker ALPHA FT-IR with a diamond ATR (400–4000 cm^{-1}). Mass spectrometry was performed either on a Jeol AccuTOF GCv (LIFDI, CI). The microanalyses were done with elementar vario MICRO cube instrument.The UV/Vis spectrum was recorded on an analytikjena Specord S 600 single beam photometer at room temperature.

[(*t*BuNAs)$_2$(*t*BuNLi)$_2$]$_2$ (1$_2$)

(tBuNAs)$_2$(tBuNH)$_2$ (2.14 g, 4.91 mmol,1.00 eq.) was dissolved in $n$pentane (70 mL) and cooled to −80 °C. Slow addition of a solution of nBuLi in $n$hexane (2.5M, 3.73 mL, 9.32 mmol, 1.90 eq.) leads to a slightly yellow solution. Afterwards, the reaction mixture was allowed to warm to room temperature and the solvent was evaporated *in vacuo*. After drying of the crude product it can be recrystallized from $n$pentane (15.0 mL) to yield **1$_2$** as a colourless crystalline solid (1.73 g, 1.93 mmol, 79%).

^{1}H NMR (300 MHz, C$_6$D$_6$): δ = 1.24 (s, 18H, As$_2$N*tBu*), 1.32 (s, 18H, AsN*tBu*Li) ppm.

^{13}C{^{1}H} NMR (75.5 MHz, C$_6$D$_6$): δ = 31.0 (s, As$_2$NC(*C*H$_3$)$_3$), 37.9 (s, N(Li)C(*C*H$_3$)$_3$), 53.9 (s, As$_2$N*C*(Me)$_3$), 54.4 (s, N(Li)*C*(Me)$_3$) ppm.

^{7}Li NMR (117 MHz, C$_6$D$_6$): δ = -2.13 ppm.

Elemental Analysis: (Found) C: 42.73, H: 7.93, N: 12.76; (calc.) C: 42.88, H: 8.10, N: 12.50.

IR: ν=2954 (m), 2858 (w), 1475 (w), 1462 (w), 1376 (w), 1357 (m), 1174 (s), 1025 (w), 917 (m), 905 (s), 767 (m), 718 (s), 687 (s), 665 (s), 545 (m), 482 (w), 469 (w), 412 (w) cm^{-1}.

[(*t*BuNAs)$_2$(*t*BuN)$_2$AsCl] (2)

1 (0.48 g, 1.06 mmol, 1.00 eq.) was dissolved in DME (10.0 mL) and cooled to −60 °C. Then $AsCl_3$ (0.19 g, 1.06 mmol, 1.00 eq.) dissolved in DME (10.0 mL) was added slowly. The solution initially turns intensively yellow but gets decolourized after the addition is complete. Afterwards, the reaction mixture was allowed to warm to room temperature and the solvent was evaporated *in vacuo*. The crude product was dissolved in toluene (20.0 mL) and filtered through a D4 frit. Concentration of the solution to 10 mL and cooling to −32 °C yields **2** as a pale yellow crystalline solid (0.36 g, 0.67 mmol, 62%).

^{1}H NMR (300 MHz, C$_6$D$_6$): δ = 1.24 (s, 9H, As$_2$N*tBu*), 1.46 (s, 27H, ClAsN*tBu* & As$_2$N*tBu*) ppm.

^{13}C{^{1}H} NMR (75.5 MHz, C$_6$D$_6$): δ = 30.6 (s, As$_2$NC(*C*H$_3$)$_3$), 32.5 (s, ClAsNC(*C*H$_3$)$_3$), 54.1 (s, As$_2$N*C*(Me)$_3$), 54.9 (s, As$_2$N*C*(Me)$_3$), 59.7 (s, ClAsN*C*(Me)$_3$) ppm.

HR-MS: CI(+) m/z = 544.02603 [M]$^{+}$; cal.= 544.02764.

Elemental Analysis: (Found) C: 35.80, H: 6.65, N: 10.39; (calc.) C: 35.28, H: 6.66, N: 10.29.

IR: ν = 2954 (m), 2862 (w), 1464 (m), 1387 (w), 1359 (m), 1237 (w), 1203 (m), 1177 (s), 1038 (w), 980 (m), 925 (m), 906 (m), 810 (s), 710 (w), 515 (w), 485 (w), 462 (w), 419 (m) cm^{-1}.

[(*t*BuNAs)$_2$(*t*BuN)$_2$SbCl] (3)[4]

1 (0.31 g, 0.69 mmol, 1.00 eq.) was dissolved in DME (10.0 mL) and cooled to −60 °C. Then $SbCl_3$ (0.16 g, 0.69 mmol, 1.00 eq.) dissolved in DME (10.0 mL) was added slowly. The solution initially turns pale yellow but gets decolourized after the addition is complete. Afterwards, the reaction mixture was allowed to warm to room temperature and the solvent was evaporated *in vacuo*. The crude product was dissolved in toluene (20.0 mL) and filtered through a D4 frit. Concentration of the solution to 8.00 mL and cooling to −32 °C yields **3** as a colourless crystalline solid (0.39 g, 0.66 mmol, 95%).

^{1}H NMR (300 MHz, C_6D_6): δ = 1.10 (s, 9H, As$_2$N*tBu*), 1.31 (s, 9H, As$_2$N*tBu*), 1.64 (s, 18H, SbN*tBu*) ppm.

^{13}C{^{1}H} NMR (75.5 MHz, C_6D_6): δ = 25.9 (s, As$_2$NC(CH$_3$)$_3$), 32.4 (s, As$_2$NC(CH$_3$)$_3$), 35.0 (s, SbNC(CH$_3$)$_3$), 54.7 (s, As$_2$NC(Me)$_3$), 55.3 (s, As$_2$NC(Me)$_3$), 59.3 (s, SbNC(Me)$_3$) ppm.

HR-MS: CI(+) m/z = 592.01034 $[M]^+$; cal.= 592.01026.

IR: ν = 2956 (m), 2945 (m), 2857 (w), 1458 (w), 1387 (w), 1359 (s), 1182 (s), 1033 (w), 968 (m), 939 (m), 789 (s), 774 (s), 750 (s), 727 (s), 598 (m), 516 (w), 495 (s), 480 (w), 413 (w) cm^{-1}.

[(*t*BuNAs)$_2$(*t*BuN)$_2$BiCl] (4)[4]

1 (0.31 g, 0.69 mmol, 1.00 eq.) was dissolved in DME (10.0 mL) and cooled to −60 °C. Then $BiCl_3$ (0.22 g, 0.69 mmol, 1.00 eq.) dissolved in DME (10.0 mL) was added slowly. Upon addition the solution turns intensively yellow. Then, the reaction mixture was allowed to warm to room temperature and the solvent was evaporated *in vacuo*. The crude product was dissolved in toluene (20.0 mL) and filtered through a D4 frit. Concentration of the solution to 8.00 mL and cooling to −32 °C yields **4** as a yellow crystalline solid (0.44 g, 0.64 mmol, 93%).

^{1}H NMR (300 MHz, C_6D_6): δ = 1.18 (s, 18H, As$_2$N*tBu*), 1.51 (s, 18H, BiN*tBu*) ppm.

^{13}C{^{1}H} NMR (75.5 MHz, C_6D_6): δ = 29.7 (s, As$_2$NC(CH$_3$)$_3$), 36.4 (s, BiNC(CH$_3$)$_3$), 54.8 (s, As$_2$NC(Me)$_3$), 58.6 (s, BiNC(Me)$_3$) ppm.

HR-MS: CI(+) m/z = 678.08387 $[M]^+$; cal.= 678.08643.

Elemental Analysis: (Found) C: 28.15, H: 5.28, N: 8.23; (calc.) C: 28.31, H: 5.35, N: 8.25.

IR: ν = 2952 (m), 2896 (w), 2858 (w), 1458 (w), 1385 (w), 1356 (m), 1262 (w), 1183 (s), 1098 (w), 1032 (w), 972 (m), 940 (m), 788 (m), 770 (m), 728 (s), 589 (m), 522 (w), 504 (w), 490 (w) , 472 (w) , 410 (w) cm^{-1}.

[(*t*BuNAs)$_2$(*t*BuN)$_2$AsP(*t*Bu)Sb*t*Bu$_2$] (5)

2 (0.13 g, 0.23 mmol 2.00 eq.) was dissolved in Et_2O (10.0mL), cooled to −40 °C and (*t*Bu$_2$SbP[Li·OEt$_2$]*t*Bu)$_2$ (92.0 mg, 0.11 mmol, 1.00 eq.) dissolved in Et_2O (10.0 mL) was added slowly. Upon addition the solution turns yellow. Then, the reaction mixture was allowed to warm to room

temperature and the solvent was evaporated *in vacuo*. After drying in a fine vacuum the crude product was dissolved in *n*pentane (10.0 mL) and filtered through a D4 frit. Concentration of the solution to 1.00 mL and cooling to −32 °C yields a mixture of compounds containing **5** as a yellow crystals.

$^{31}P\{^{1}H\}$ NMR (101 MHz, $C_{6k}D_6$): δ = 61.7 (s, As(Sb)*Pt*Bu) ppm.

^{31}P NMR (101 MHz, C_6D_6): δ = 61.7 (m, As(Sb)*Pt*Bu) ppm.

[(*t*BuNAs)$_2$(*t*BuN)$_2$SbP(*t*Bu)Sb*t*Bu$_2$] (6)

3 (0.13 g, 0.21 mmol 2.00 eq.) was dissolved in Et_2O (10.0mL), cooled to −40 °C and (*t*Bu$_2$SbP[Li·OEt$_2$]*t*Bu)$_2$ (86.0 mg, 0.11 mmol, 1.00 eq.) dissolved in Et_2O (10.0 mL) was added slowly. Upon addition the solution turns yellow. Then, the reaction mixture was allowed to warm to room temperature and the solvent was evaporated *in vacuo*. After drying in a fine vacuum the crude product was dissolved in *n*pentane (10.0 mL) and filtered through a D4 frit. Concentration of the solution to 1.00 mL and cooling to −32 °C yields **6** as a yellow crystalline solid (0.11 g, 0.13 mmol, 62 %).

^{1}H NMR (300 MHz, C_6D_6): δ = 1.29 (s, 9H, As$_2$N*tBu*), 1.54 (s, 9H, As$_2$N*tBu*), 1.59 (s, 18H, Sb(*tBu*)$_2$), 1.68 (s, 18H, Sb(As)N*tBu*), 1.77 (d, $^{3}J_{PH}$ = 11.71 Hz, 9H, P*tBu*) ppm.

$^{13}C\{^{1}H\}$ NMR (75.5 MHz, C_6D_6): δ = 30.4 (d, $^{4}J_{CP}$ = 5.0 Hz, As$_2$NC(CH$_3$)$_3$), 31.8 (s, As$_2$NC(CH$_3$)$_3$), 32.9 (d, $^{2}J_{CP}$ = 4.6 Hz, SbC(Me)$_3$), 34.4 (d, $^{3}J_{CP}$ = 2.0 Hz, SbC(CH$_3$)$_3$), 35.7 (d, $^{4}J_{CP}$ = 2.8 Hz, SbNC(CH$_3$)$_3$), 36.4 (d, $^{2}J_{CP}$ = 11.8 Hz, PC(CH$_3$)$_3$), 39.0 (d, $^{1}J_{CP}$ = 52.2 Hz, PC(CH$_3$)$_3$), 54.9 (s, As$_2$NC(CH$_3$)$_3$), 55.7 (s, As$_2$NC(CH$_3$)$_3$), 59.1 (s, SbNC(CH$_3$)$_3$) ppm.

$^{31}P\{^{1}H\}$ NMR (101 MHz, $C_{6k}D_6$): δ = 22.5 (s, Sb(Sb)*Pt*Bu) ppm.

^{31}P NMR (101 MHz, C_6D_6): δ = 22.5 (m, Sb(Sb)*Pt*Bu) ppm.

HR-MS: LIFDI(+) m/z = 555.04370 [M-*t*BuPSb*t*Bu$_2$]$^+$, cal.= 555.04101; 323.09032 [*t*BuPSb*t*Bu$_2$]$^+$, cal.= 323.08886.

Elemental Analysis: (Found) C: 37.67, H: 6.70, N: 6.73; (calc.) C: 38.21, H: 7.21, N: 6.37.

IR: ν = 2959 (m), 2923 (w), 2887 (w), 2848 (w), 1459 (w), 1385 (w), 1359 (m), 1260 (s), 1180 (w), 1148 (w), 1093 (s), 1018 (s), 921 (w), 863 (w), 799 (s), 754 (w), 694 (w) cm^{-1}.

[(*t*BuNAs)$_2$(*t*BuN)$_2$BiP(*t*Bu)Sb*t*Bu$_2$] (7)

4 (0.25 g, 0.37 mmol 2.00 eq.) was dissolved in Et_2O (10.0mL), cooled to −40 °C and (*t*Bu$_2$SbP[Li·OEt$_2$]*t*Bu)$_2$ (0.15 g, 0.19 mmol, 1.00 eq.) dissolved in Et_2O (7.5 mL) was added slowly. Upon addition the solution turns intensively red. Then, the reaction mixture was allowed to warm to room temperature and the solvent was evaporated *in vacuo*. The crude product was dissolved in *n*pentane (10.0 mL) and filtered through a D4 frit. Concentration of the solution to 1.00 mL and cooling to −32 °C yields **7** as a red crystalline solid (0.18 g, 0.19 mmol, 51 %).

^{1}H NMR (300 MHz, C_6D_6): δ = 1.30 (s, 18H, As$_2$N*tBu*), 1.56 (s, 18H, Bi(As)N*tBu*)), 1.59 (s, 18H, Sb(*tBu*)$_2$), 2.01 (d, $^{3}J_{PH}$ = 11.98 Hz, 9H, P*tBu*) ppm.

$^{13}C\{^{1}H\}$ NMR (75.5 MHz, C_6D_6): δ = 31.0 (s, As$_2$NC(CH$_3$)$_3$), 31.2 (s, As$_2$NC(CH$_3$)$_3$), 33.7 (d, $^{2}J_{CP}$ = 5.2 Hz, SbC(Me)$_3$), 34.4 (d, $^{3}J_{CP}$ = 2.1 Hz, SbC(CH$_3$)$_3$), 37.7 (d, $^{4}J_{CP}$ = 5.3 Hz, BiNC(CH$_3$)$_3$), 38.7 (d, $^{2}J_{CP}$ = 50.2 Hz,

PC(CH_3)$_3$), 39.6 (d, $^1J_{CP}$ = 11.4 Hz, PC(CH$_3$)$_3$), 54.9 (s, As_2NC(CH_3)$_3$), 55.0 (s, As_2NC(CH_3)$_3$), 55.4 (s, As_2NC(CH_3)$_3$), 59.2 (s, BiNC(CH_3)$_3$) ppm.

^{31}P{^{1}H} NMR (101 MHz, $C_{6k}D_6$): δ = 37.6 (s, Bi(Sb)*Pt*Bu) ppm.

^{31}P NMR (101 MHz, C_6D_6): δ = 37.6 (m, Bi(Sb)*Pt*Bu) ppm.

HR-MS: CI(-) m/z = 643.11975 [M-*t*BuPSb*t*Bu$_2$]$^-$, cal.= 643.11757; 323.08859 [*t*BuPSb*t*Bu$_2$]$^-$, cal.= 323.08886.

Elemental Analysis: (Found) C: 34.51, H: 6.46, N: 5.51; (calc.) C: 34.76, H: 6.56, N: 5.79.

IR: ν = 2950 (m), 2919 (m), 2883 (m), 2845 (m), 1455 (m), 1384 (m), 1356 (s), 1213 (m), 1177 (s), 1146 (m), 1029 (w), 950 (m), 915 (w), 784 (m), 758 (m), 716 (s), 515 (w), 480 (w), 408 (w) cm^{-1}.

3 X-ray Structure Analysis

The data were collected on a STOE IPDS 2 or STOE IPDS 2T diffractometer. The radiation used was monochromatic Mo-Kα. The solution of the structure was performed with intrinsic phasing with the SHELXT-2015 solution program while for the structure refinement with full-matrix least-squares against F^2 the SHELXL-2015 package was used, both within the OLEX² environment.[5–7]

Crystal Data of $\mathbf{1_2}$:

$C_{32}H_{72}As_4Li_4N_8$, 896.41 g mol^{-1}, T = 100 K, monoclinic, $C2/c$, a = 23.964(1) Å, b = 9.7412(5) Å, c = 24.158(1) Å, β = 131.130(3), V = 4247.6(4) Å^3, Z = 4, ρ = 1.402 g cm^{-3}, μ = 3.151 mm^{-1}, F(000) = 1856, GooF = 1.036. A total of 12220 reflections was collected, of which 4165 were unique (R(int) = 0.0361). R_1 (wR_2 all data) = 0.0326 (0.0758) for 229 parameters and 3390 reflections ($I > 2\sigma(I)$). CCDC 1983180.

Crystal Data of 1·DME:

$C_{20}H_{46}As_2Li_2N_4O_2$, 538.33g mol^{-1}, T = 100 K, monoclinic, $P2_1/n$, a = 9.9039(5) Å, b = 18.8433(6) Å, c = 15.3740(7) Å, β = 90.020(4), V = 2869.1(2) Å^3, Z = 4, ρ = 1.246 g cm^{-3}, μ = 2.349 mm^{-1}, F(000) = 1128, GooF = 1.289. A total of 29778 reflections was collected, of which 7275 were unique (R(int) = 0.0290). R_1 (wR_2 all data) = 0.0695 (0.1753) for 385 parameters and 6354 reflections ($I > 2\sigma(I)$). CCDC 1983179.

Crystal Data of 2:

$C_{16}H_{36}As_3ClN_4$, 544.70 g mol^{-1}, T = 100 K, monoclinic, $P2_1/m$, a = 9.778(2) Å, b = 11.712(2) Å, c = 9.986(2) Å, β = 97.792(16), V = 1133.0(4) Å^3, Z = 2, ρ = 1.597 g cm^{-3}, μ = 4.523 mm^{-1}, F(000) = 552, GooF = 1.079. A total of 7619 reflections was collected, of which 2332 were unique (R(int) = 0.0294). R_1 (wR_2 all data) = 0.0290 (0.0744) for 126 parameters and 2104 reflections ($I > 2\sigma(I)$). CCDC 1983177.

Crystal Data of 4 ·$^1/_2$ toluene:

$C_{39}H_{80}As_4Bi_2Cl_2N_8$, 1449.65 g mol^{-1}, T = 100 K, triclinic, $P\overline{1}$, a = 9.5377(5) Å, b = 11.9645(7) c = 13.1596(8) Å, α = 111.668(4), β = 108.299(4), γ = 90.013(4), V = 1313.5(1) Å^3, Z = 1, ρ = 1.833 g cm^{-3}, μ = 9.326 mm^{-1}, F(000) = 702, GooF = 1.056. A total of 41070 reflections was collected, of which 17736 were unique (R(int) = 0.0272). R_1 (wR_2 all data) = 0.0537 (0.1491) for 245 parameters and 16549 reflections ($I > 2\sigma(I)$). CCDC 1983182.

Crystal Data of [(*t*BuNAs)$_2$(*t*BuN)$_2$AsP(*t*Bu)Sb*t*Bu$_2$] (5):

$C_{28}H_{63}As_3N_4PSb$, 833.34 g mol^{-1}, T = 100 K, Mo-Kα radiation, monoclinic, Cc, a = 25.558(2) Å, b = 14.669(1) Å, c = 21.036(2) Å, β = 109.241(5)°, V = 7446(1) Å^3, Z = 8, ρ = 1.487 g cm^{-3}, μ = 3.452 mm^{-1}, F(000) = 3392, GooF = 0.985. A total of 23776 reflections was collected, of which 13230 were unique (R(int) = 0.0955). R_1 (wR_2 all data) = 0.0890 (0.2211) for 650 parameters and 7986 reflections ($I > 2\sigma(I)$). Flack parameter = -0.008(5). CCDC 1983178.

Crystal Data of 6:

$C_{28}H_{63}As_2N_4PSb_2$, 880.13 g mol^{-1}, T = 100 K, Mo-Kα radiation, monoclinic, Cc, a = 25.216(5) Å, b = 14.794(3) Å, c = 21.240(4) Å, β = 108.31(3)°, V = 7522(3) Å^3, Z = 8, ρ = 1.554 g cm^{-3}, μ = 3.247 mm^{-1}, F(000) = 3536, GooF = 1.000. A total of 49008 reflections was collected, of which 14632 were unique (R(int) = 0.0230). R_1 (wR_2 all data) = 0.0240 (0.0538) for 709 parameters and 13510 reflections ($I > 2\sigma(I)$). Flack parameter = -0.014(6). CCDC 1983184.

Crystal Data of 7:

$C_{28}H_{63}As_2BiN_4PSb$, 967.36 g mol^{-1}, T = 100 K, triclinic, $P\overline{1}$, a = 9.9436(7) Å, b = 13.3807(8) Å, c = 15.7597(11) Å, α = 100.892(5)°, β = 105.489(6)°, γ = 103.984(5)°, V = 1887.4(2) Å^3, Z = 2, ρ = 1.702 g cm^{-3}, μ = 7.175 mm^{-1}, F(000) = 948, GooF = 0.954. A total of 33940 reflections was collected, of which 7401 were unique (R(int) = 0.0446). R_1 (wR_2 all data) = 0.0455 (0.1121) for 355 parameters and 5331 reflections ($I > 2\sigma(I)$). CCDC 1983183.

Crystal Data of [$As_{1.09}$(N*t*Bu)$_2$$P_{0.91}$](*t*BuN)$_2$AsCl:

$C_{16}H_{36}As_{2.09}ClN_4P_{0.91}$, 504.48 g mol^{-1}, T = 100 K, monoclinic, $P2_1/m$, a = 9.716(2) Å, b = 11.744(2) Å, c = 9.935(2) Å, β = 97.89(3), V = 1123.0(4) Å^3, Z = 2, ρ = 1.506 g cm^{-3}, μ = 3.291 mm^{-1}, F(000) = 528, GooF = 1.097. A total of 8050 reflections was collected, of which 2316 were unique (R(int) = 0.0202). R_1 (wR_2 all data) = 0.0230 (0.0576) for 136 parameters and 2084 reflections ($I > 2\sigma(I)$). CCDC 1983181.

4 UV-Vis Spectra, measured and calculated

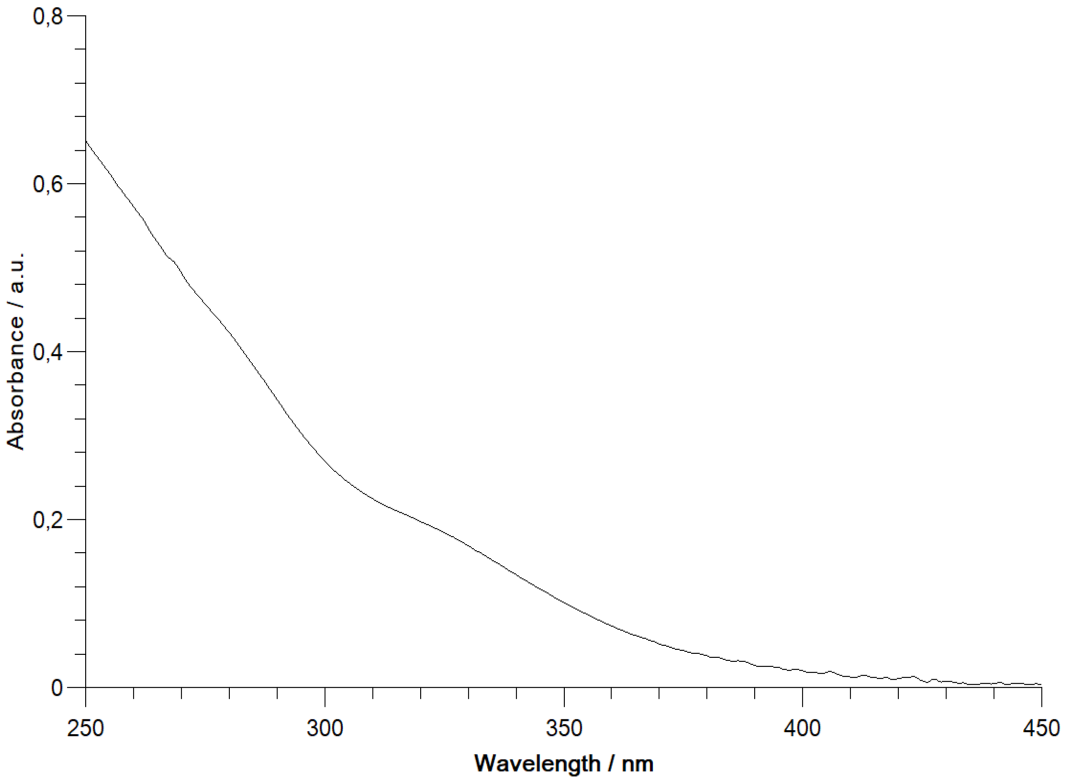

Figure S3. UV-Vis-Spectra of **6** in *n*heptane.

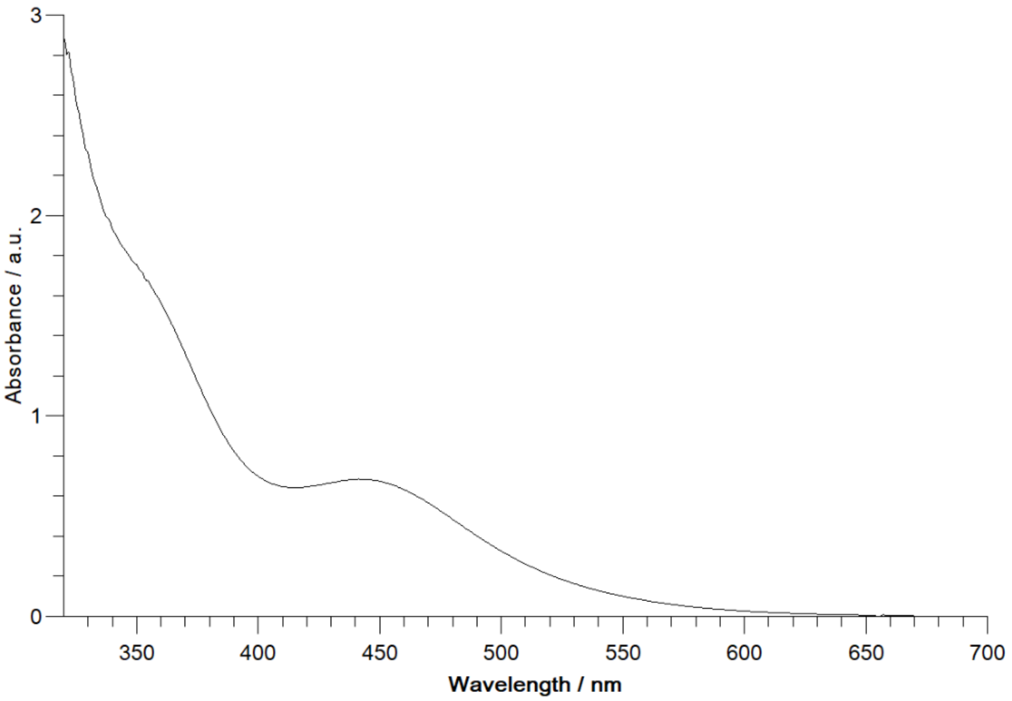

Figure S4. UV-Vis-Spectra of **7** in toluene.

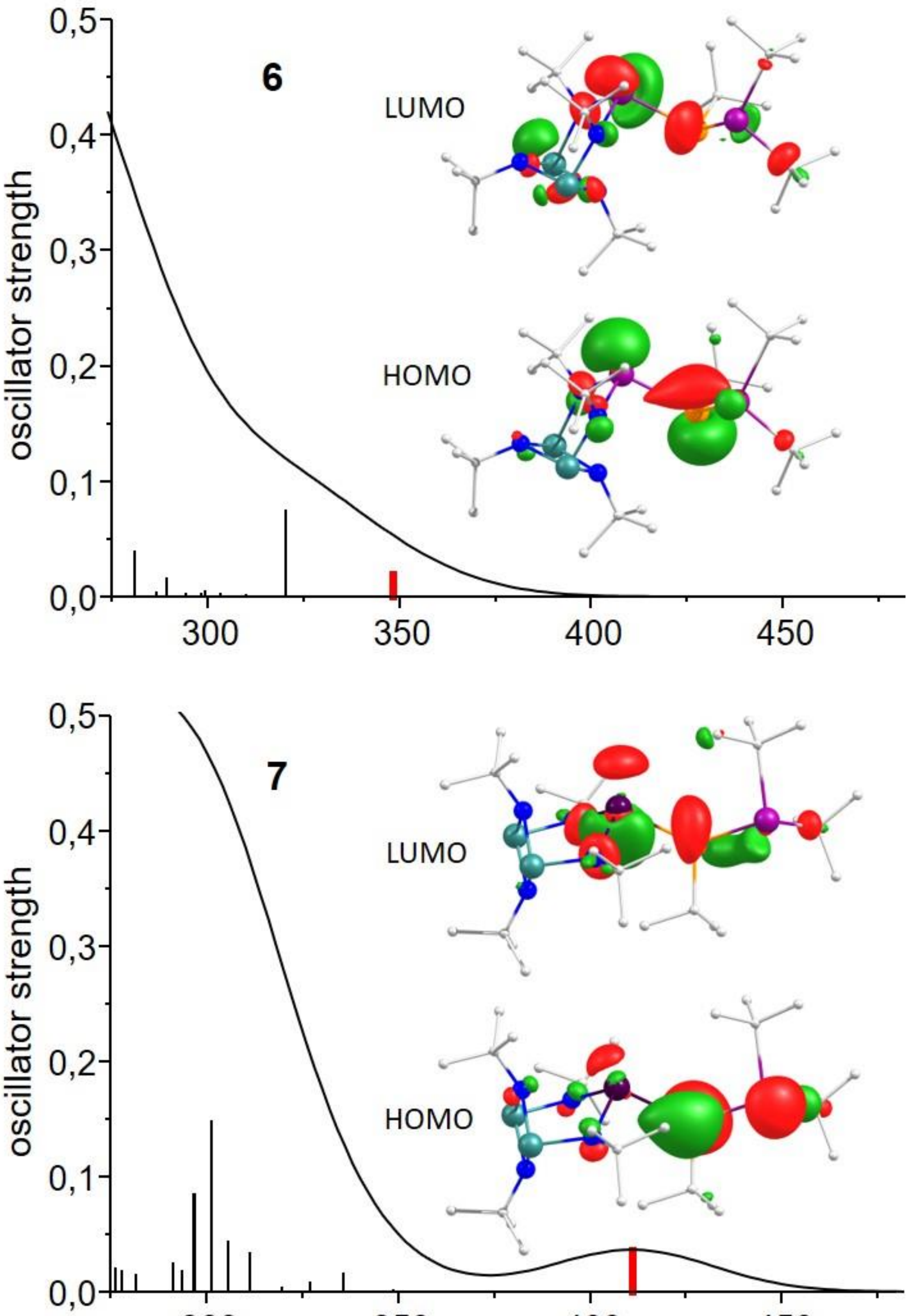

Figure S5. Calculated excitation energies and simulated spectra (FWHM = 50 nm) for compounds **6** and **7**. The first excitation peak is marked in red. In both cases it is a transition from the HOMO to the LUMO, which are plotted with iso-surfaces drawn at 0.05 a.u. For details concerning the DFT calculations, see main text.

5 Mass Spectra

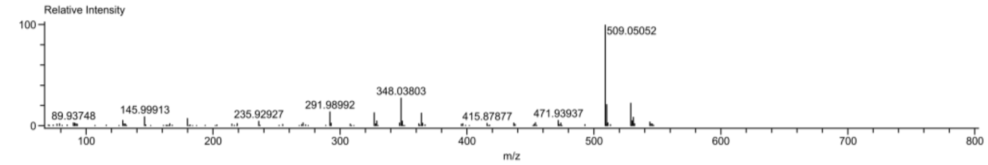

Figure S6. CI(+)-MS of **2.**

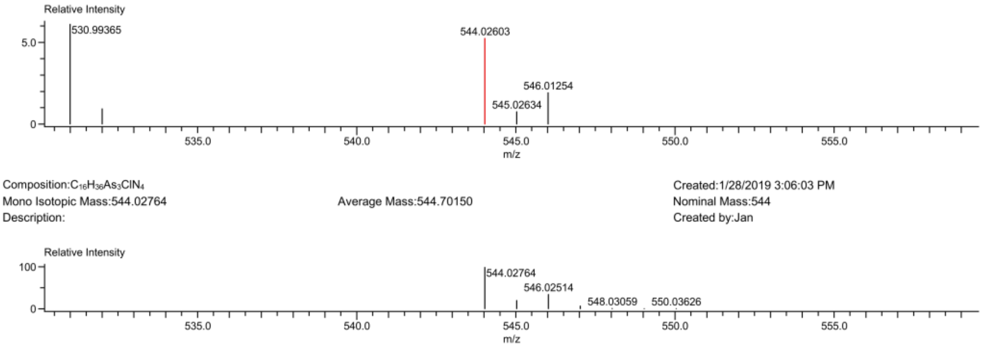

Mass	Intensity	Relative Intensity	Calc. Mass	Mass Difference [mDa]	Mass Difference [ppm]	Possible Formula	Unsaturation Number
544.02603	2008.51	5.24	544.02764	-1.60	-2.95	$^{12}C_{16}{}^{1}H_{36}{}^{75}As_{3}{}^{35}Cl_{1}{}^{14}N_{4}$	2.0

Figure S7. HR-CI(+)-MS of **2** as $[M]^+$ (top) and simulation of the corresponding sum formula (bottom).

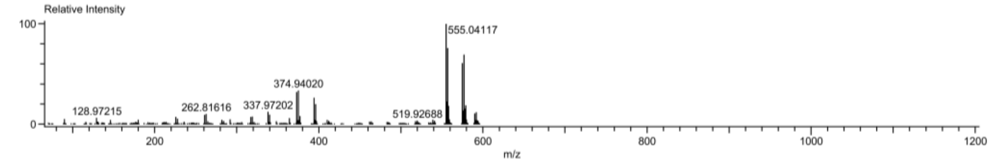

Figure S8. CI(+)-MS of **3.**

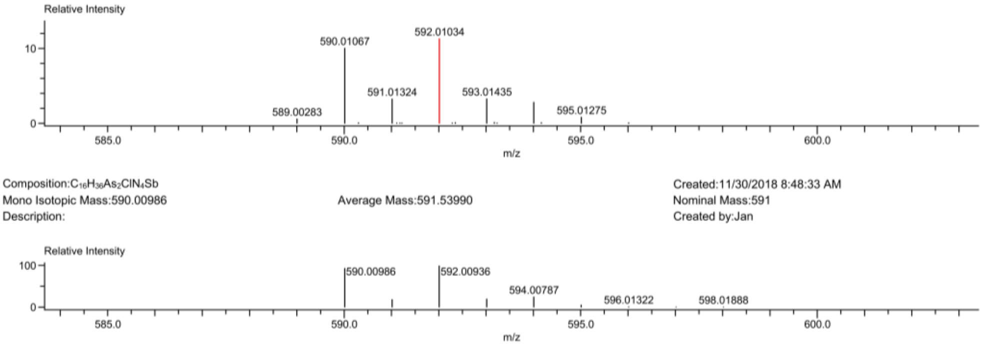

Mass	Intensity	Relative Intensity	Calc. Mass	Mass Difference [mDa]	Mass Difference [ppm]	Possible Formula	Unsaturation Number
592.01034	43261.02	11.26	592.01026	0.08	0.14	$^{12}C_{16}{}^{1}H_{36}{}^{75}As_{2}{}^{35}Cl_{1}{}^{14}N_{4}{}^{123}Sb_{1}$	2.0

Figure S9. HR-CI(+)-MS of **3** as $[M]^+$ (top) and simulation of the corresponding sum formula (bottom).

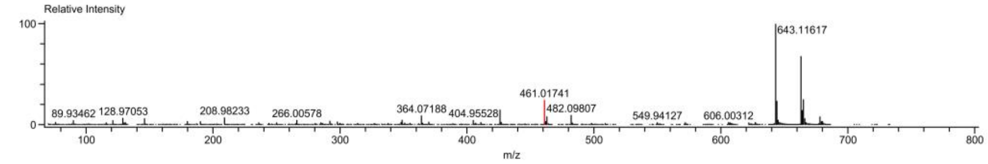

Figure S10. CI(+)-MS of **4**.

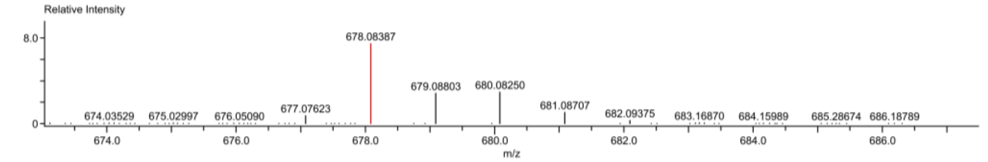

Composition:$C_{16}H_{36}As_2BiClN_4$
Mono Isotopic Mass:678.08643
Description:

Average Mass:678.76028

Created:3/7/2019 3:07:07 PM
Nominal Mass:678
Created by:

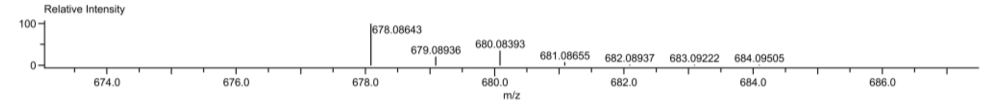

Mass	Intensity	Relative Intensity	Calc. Mass	Mass Difference [mDa]	Mass Difference [ppm]	Possible Formula	Unsaturation Number
678.08387	370688.32	7.46	678.08643	-2.55	-3.77	$^{12}C_{16}{}^{1}H_{36}{}^{75}As_2{}^{209}Bi_1{}^{35}Cl_1{}^{14}N_4$	2.0

Figure S11. HR-CI(+)-MS of **4** as $[M]^+$ (top) and simulation of the corresponding sum formula (bottom).

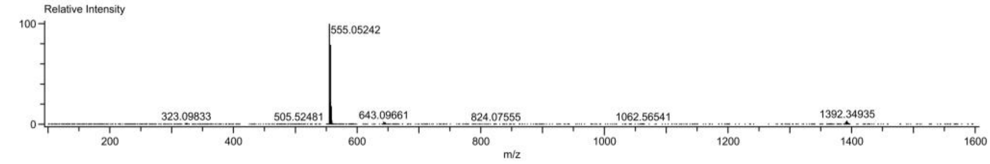

Figure S12. LIFDI(+)-MS of **6**.

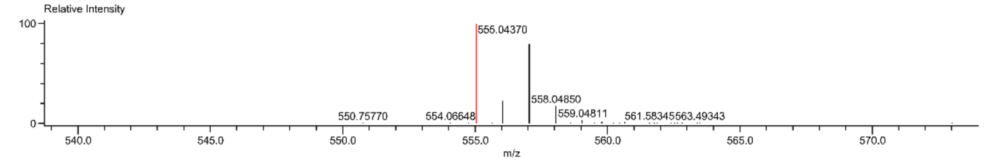

Composition:$C_{16}H_{36}As_2N_4Sb$
Mono Isotopic Mass:555.04101
Description:

Average Mass:556.08720

Created:8/8/2019 11:18:46 AM
Nominal Mass:556
Created by:Jan

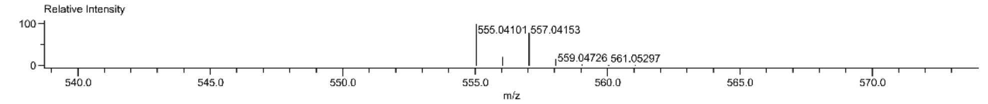

Mass	Intensity	Relative Intensity	Calc. Mass	Mass Difference [mDa]	Mass Difference [ppm]	Possible Formula	Unsaturation Number
555.04370	748978.50	100.00	555.04101	2.69	4.84	$^{12}C_{16}{}^{1}H_{36}{}^{75}As_2{}^{14}N_4{}^{121}Sb_1$	2.5

Figure S13. HR-LIFDI(+)-MS of **6** as $[M\text{-}t\text{BuPSb}t\text{Bu}_2]^+$ (top) and simulation of the corresponding sum formula (bottom).

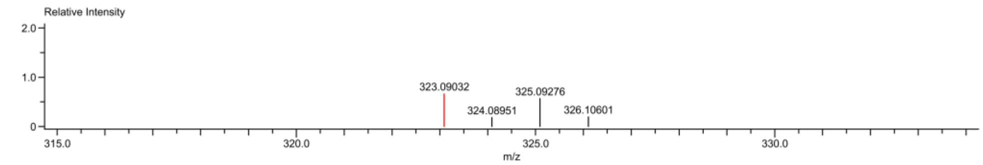

Composition:$C_{12}H_{27}PSb$
Mono Isotopic Mass:323.08886
Description:
Average Mass:324.07654
Created:8/8/2019 11:16:36 AM
Nominal Mass:324
Created by:Jan

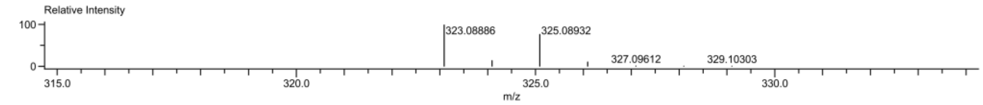

Mass	Intensity	Relative Intensity	Calc. Mass	Mass Difference [mDa]	Mass Difference [ppm]	Possible Formula	Unsaturation Number
323.09032	4800.83	0.66	323.08886	1.46	4.53	$^{12}C_{12}{}^{1}H_{27}{}^{31}P_{1}{}^{121}Sb_{1}$	1.5

Figure S14. HR-LIFDI(+)-MS of [*t*BuPSb*t*Bu$_2$]$^+$ (top) and simulation of the corresponding sum formula (bottom).

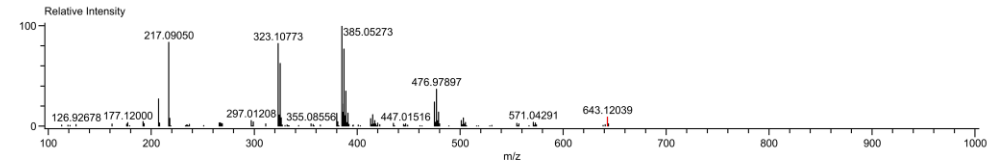

Figure S15. CI(-)-MS of **7**.

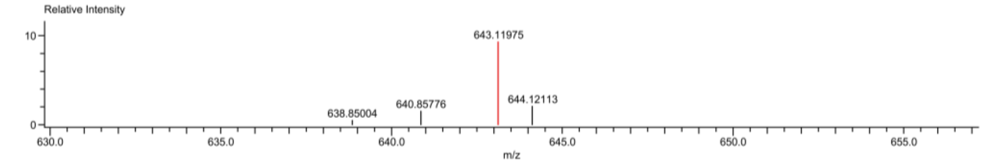

Composition:$C_{16}H_{36}As_2BiN_4$
Mono Isotopic Mass:643.11757
Description:
Average Mass:643.30758
Created:8/8/2019 11:29:08 AM
Nominal Mass:643
Created by:Jan

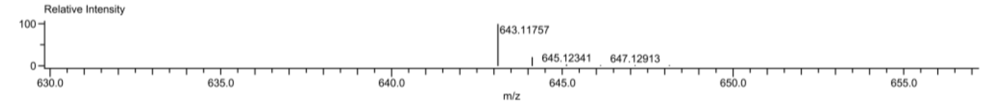

Mass	Intensity	Relative Intensity	Calc. Mass	Mass Difference [mDa]	Mass Difference [ppm]	Possible Formula	Unsaturation Number
643.11975	6772.29	9.28	643.11757	2.18	3.39	$^{12}C_{16}{}^{1}H_{36}{}^{75}As_{2}{}^{209}Bi_{1}{}^{14}N_{4}$	2.5

Figure S16. HR-CI(-)-MS of **7** as [M-*t*BuPSb*t*Bu$_2$]$^-$ (top) and simulation of the corresponding sum formula (bottom).

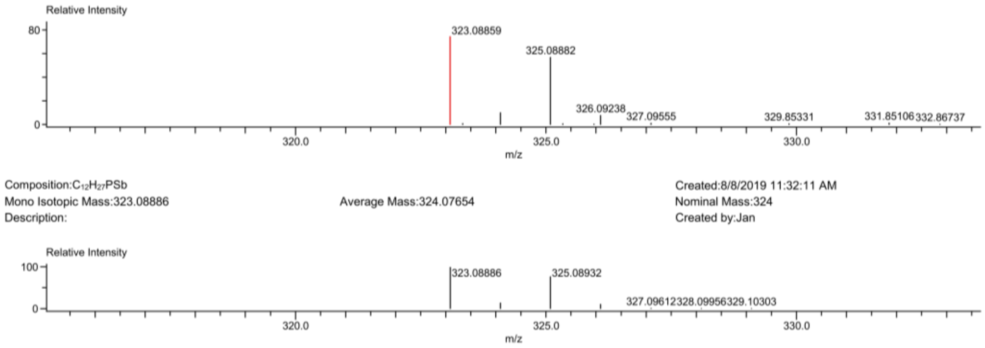

Mass	Intensity	Relative Intensity	Calc. Mass	Mass Difference [mDa]	Mass Difference [ppm]	Possible Formula	Unsaturation Number
323.08859	60150.95	74.18	323.08886	-0.27	-0.84	$^{12}C_{12}{}^{1}H_{27}{}^{31}P_{1}{}^{121}Sb_{1}$	1.5

Figure S17. HR-CI(-)-MS of [*t*BuPSb*t*Bu$_2$]$^-$ (top) and simulation of the corresponding sum formula (bottom).

6 NMR Spectra

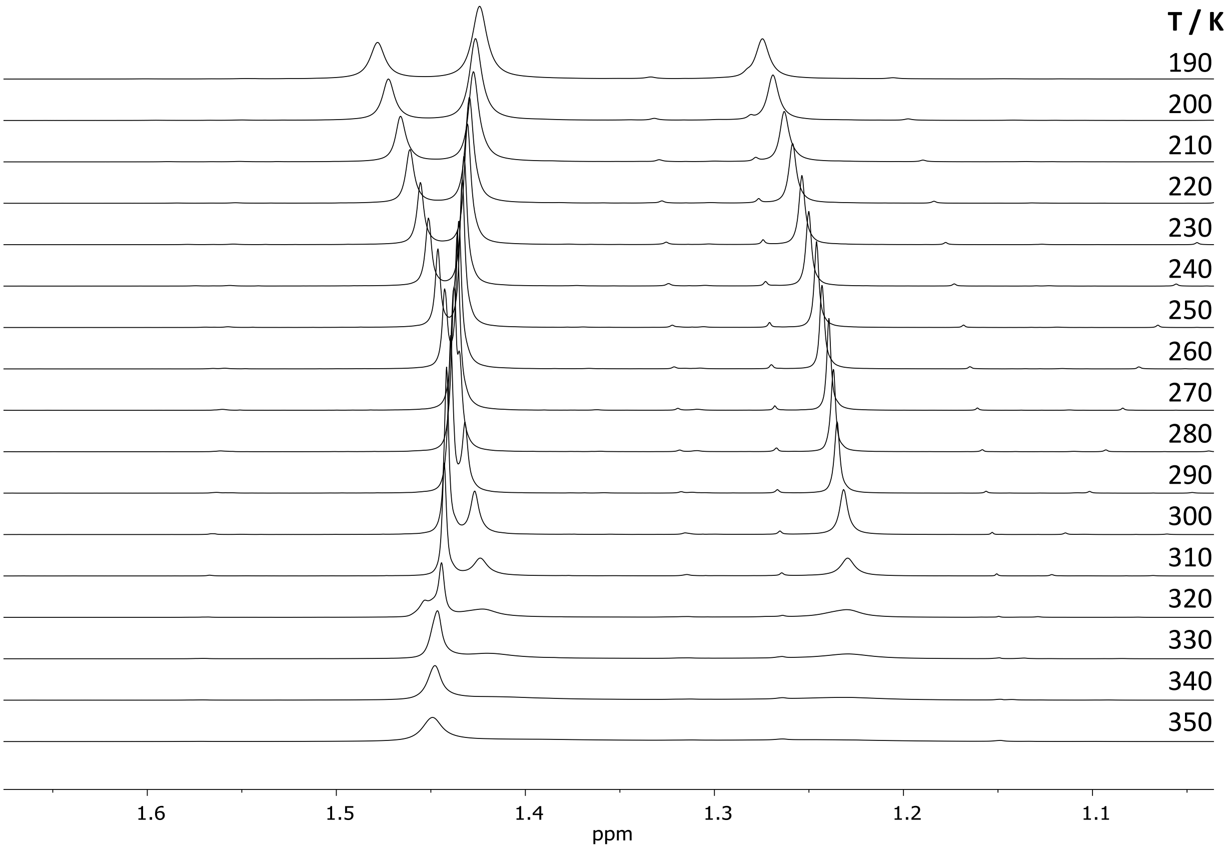

Figure S18. VT-^{1}H NMR spectrum of **2** (500 MHz, toluene-*d*^{8}).

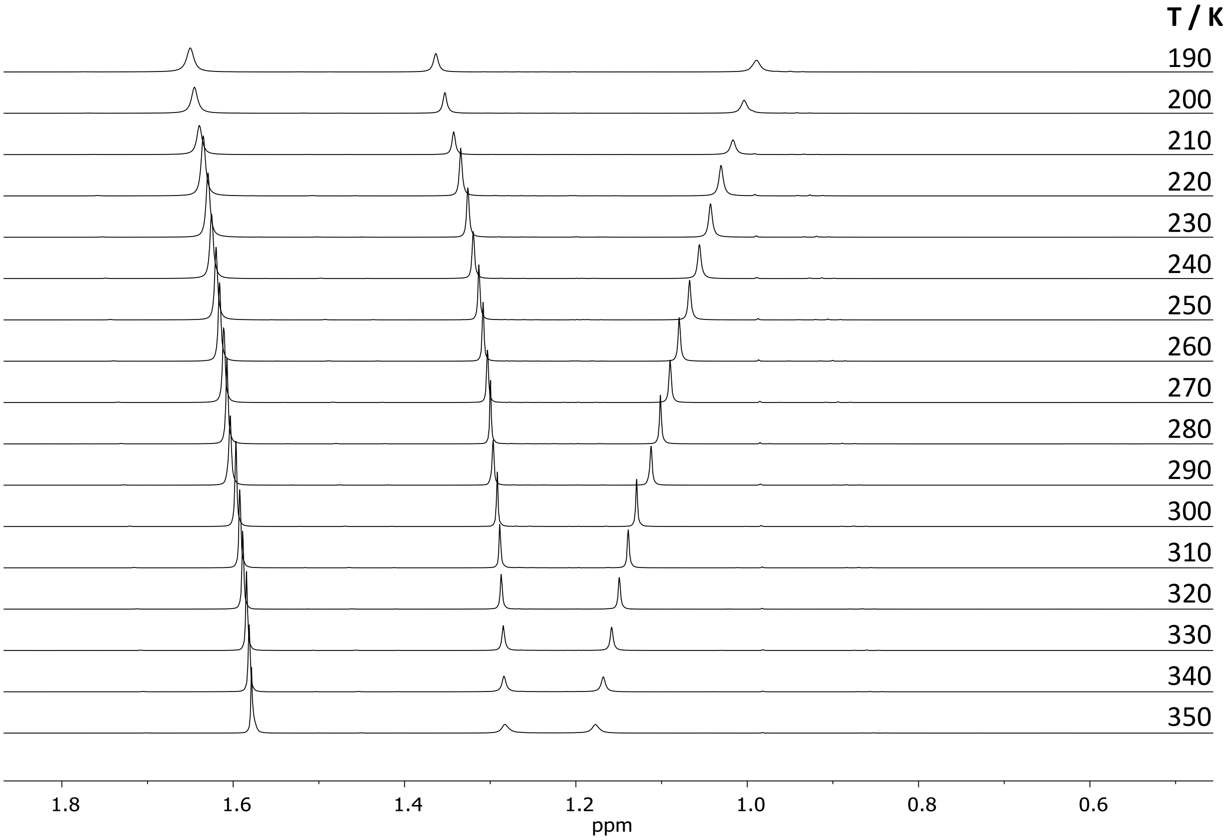

Figure S19. VT-^{1}H NMR spectrum of **3** (500 MHz, toluene-*d*^{8}).

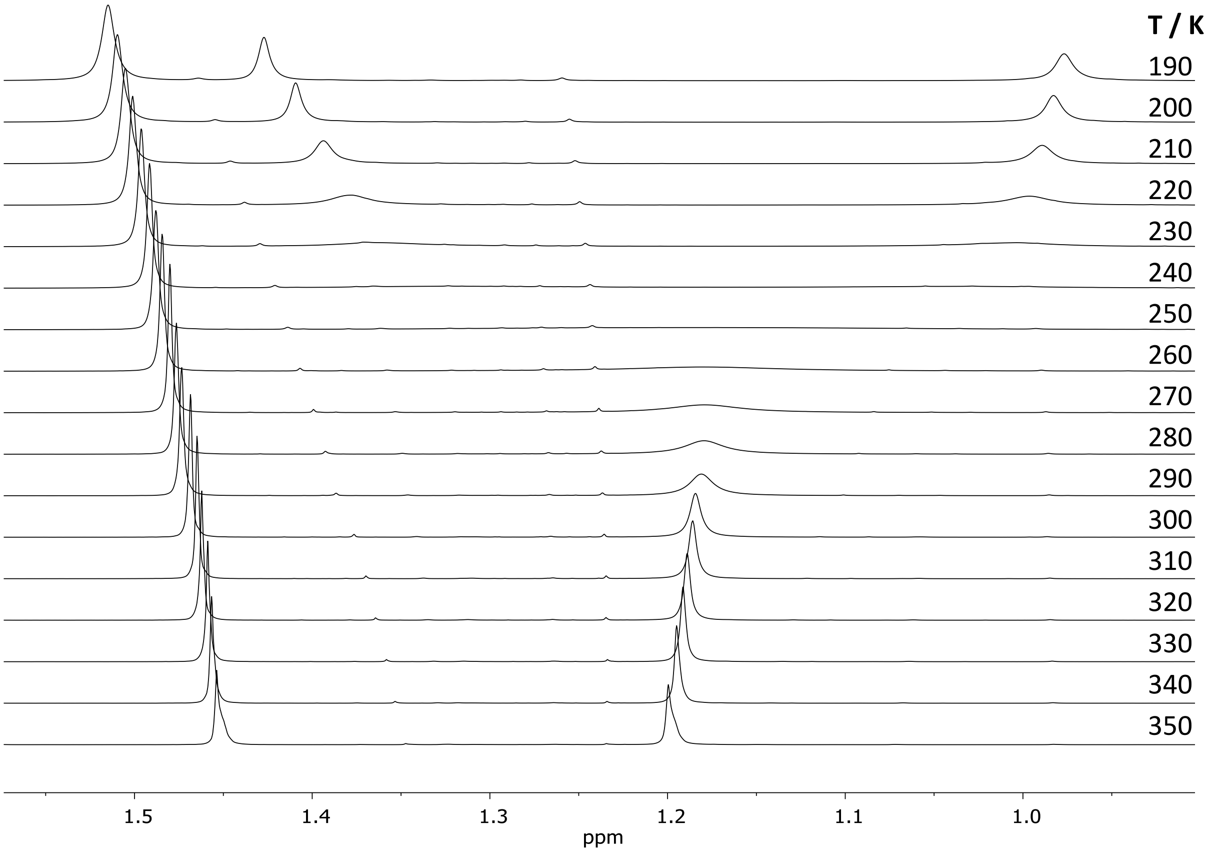

Figure S20. VT-^{1}H NMR spectrum of **4** (500 MHz, toluene-d^8).

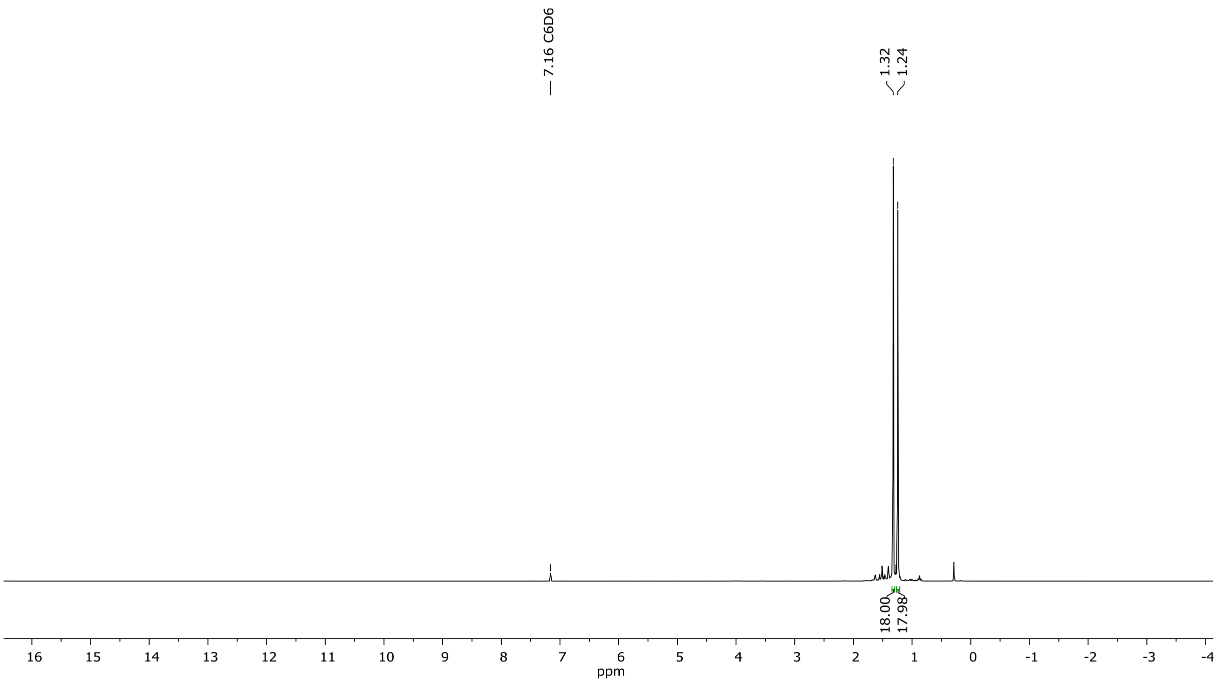

Figure S21. ^{1}H NMR spectrum of **1** (300 MHz, C_6D_6, 300K).

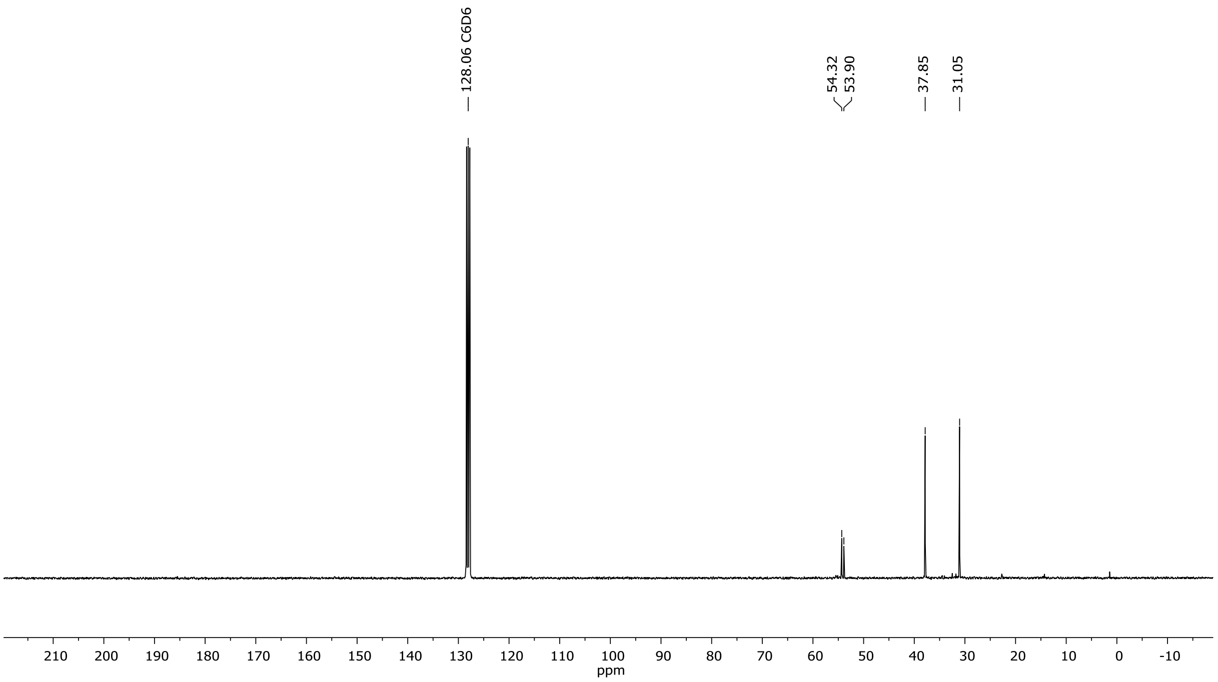

Figure S22. ^{13}C NMR spectrum of **1** (75.5 MHz, C_6D_6, 300K).

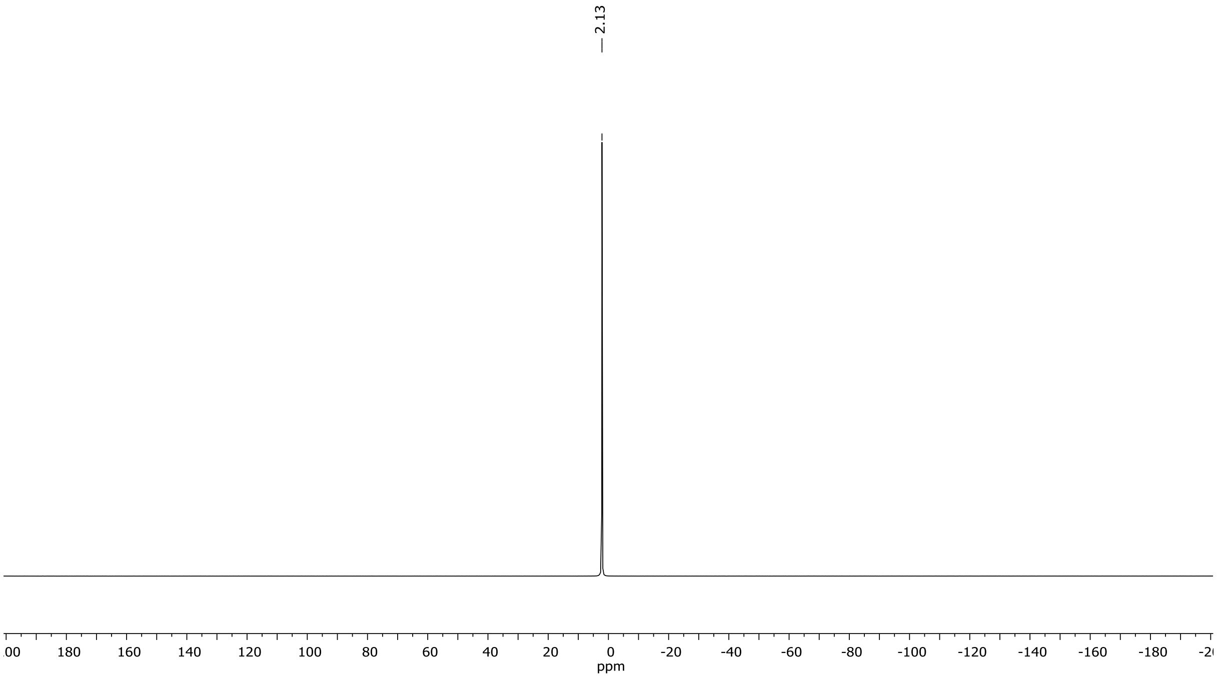

Figure S23. ^{7}Li NMR spectrum of **1** (117 MHz, C_6D_6, 300K).

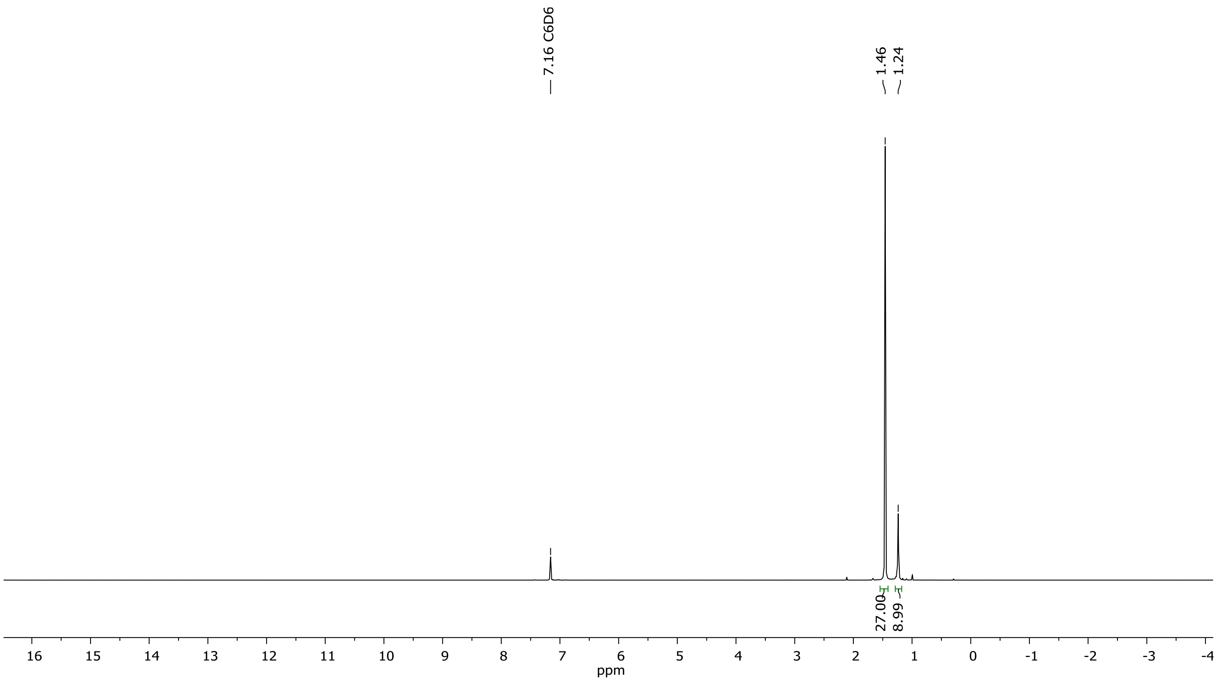

Figure S24. ^{1}H NMR spectrum of **2** (300 MHz, C_6D_6, 300K).

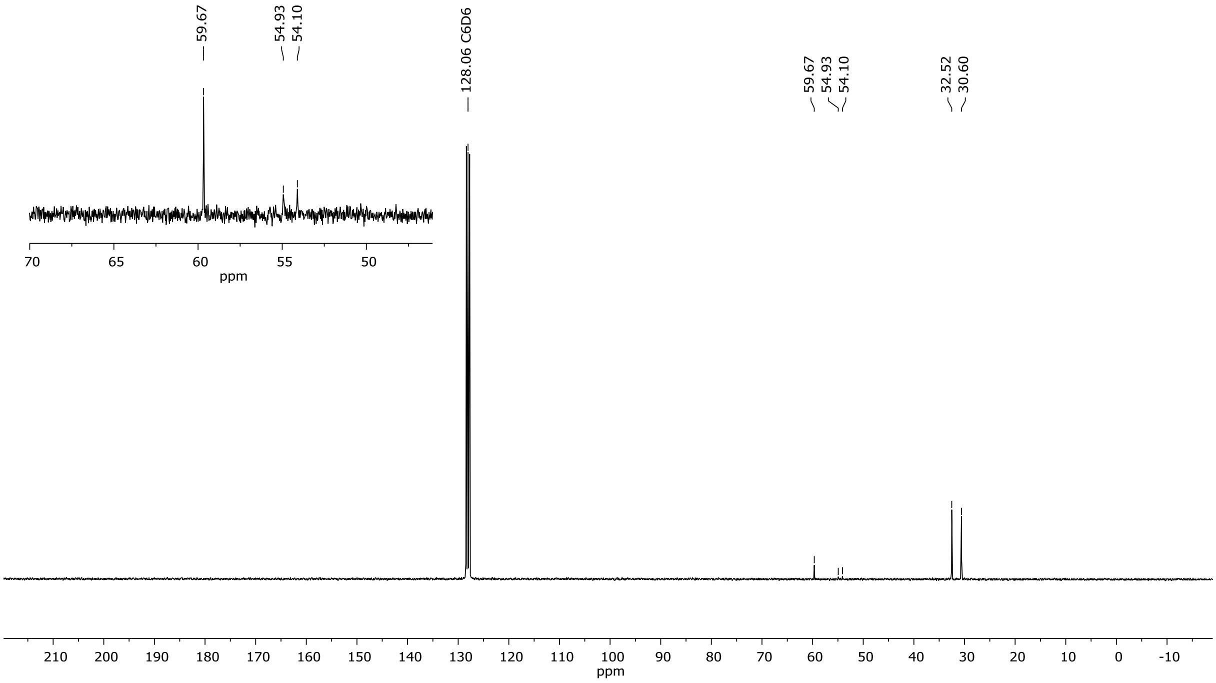

Figure S25. ^{13}C NMR spectrum of **2** (75.5 MHz, C_6D_6, 300K).

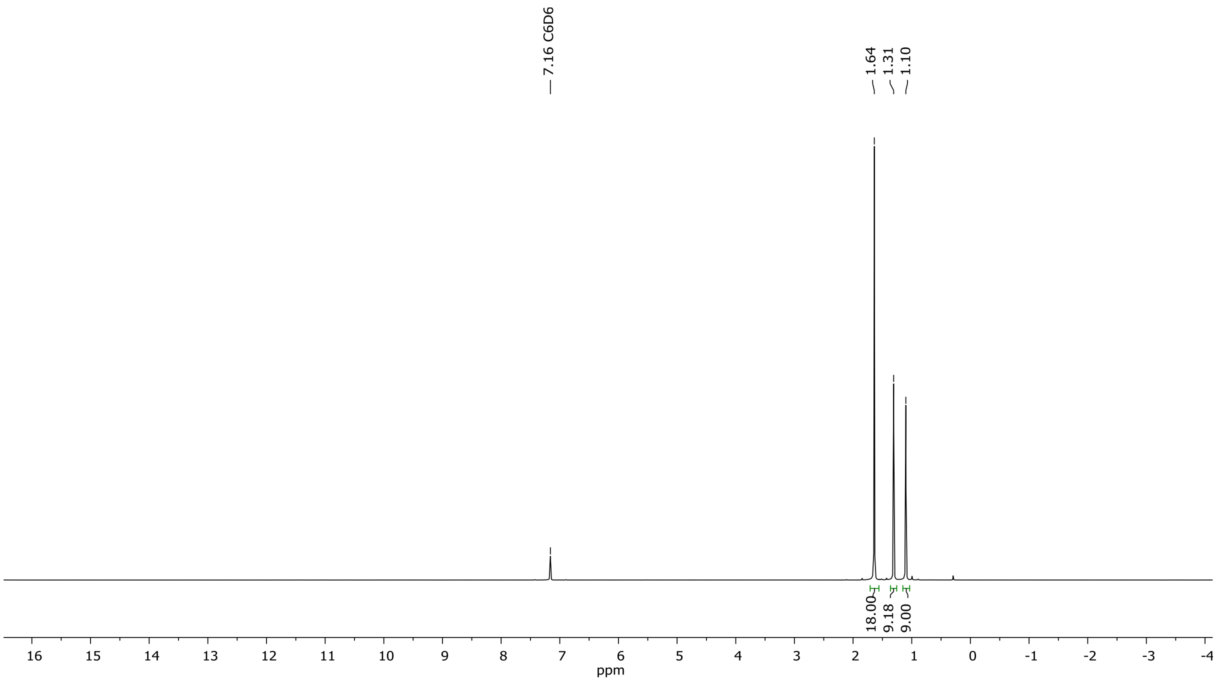

Figure S26. ^{1}H NMR spectrum of **3** (300 MHz, C_6D_6, 300K).

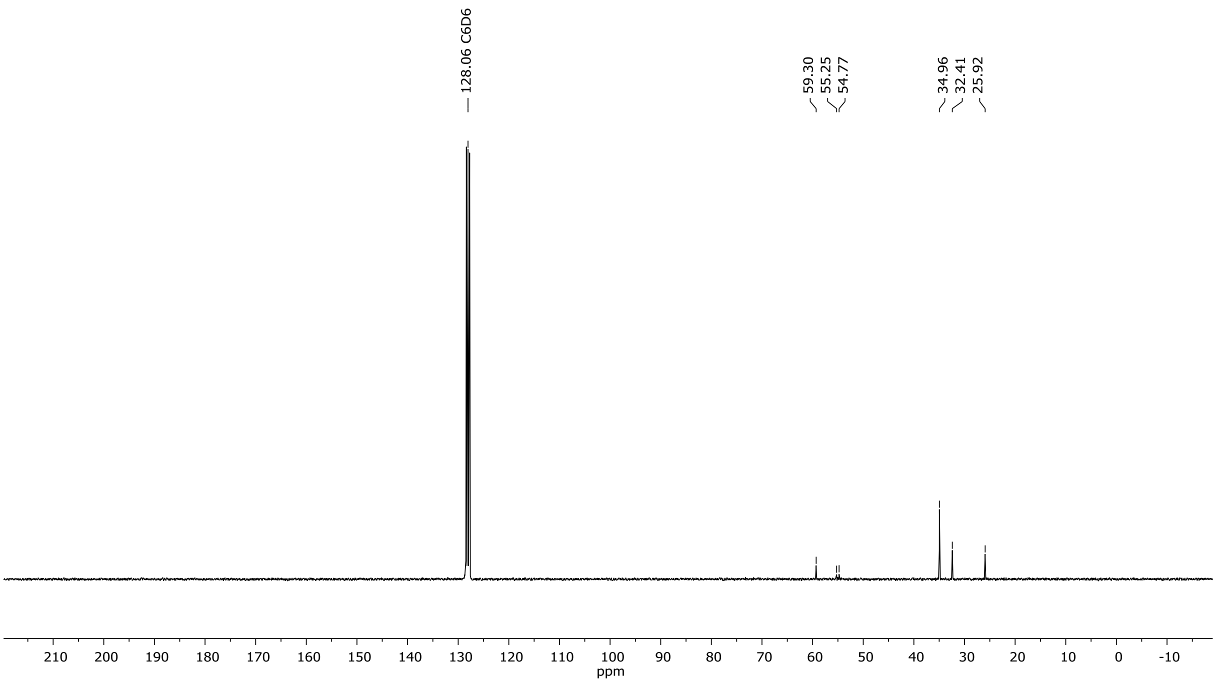

Figure S27. ^{13}C NMR spectrum of **3** (75.5 MHz, C_6D_6, 300K).

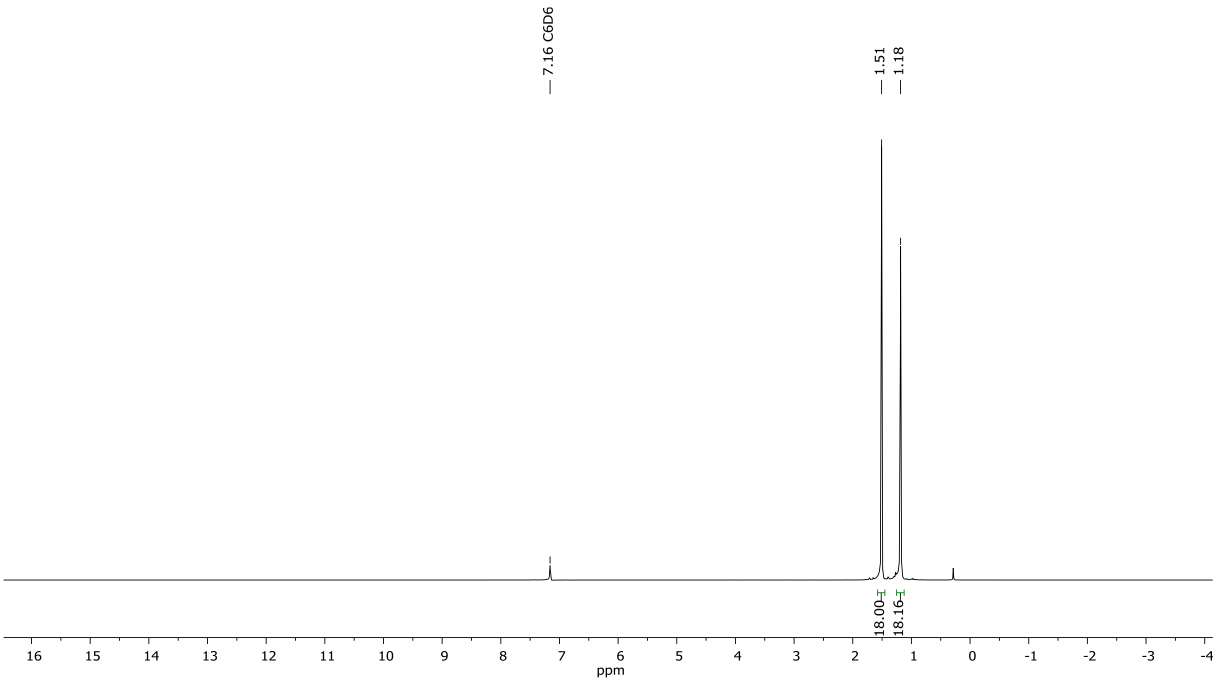

Figure S28. ^{1}H NMR spectrum of **4** (300 MHz, C_6D_6, 300K).

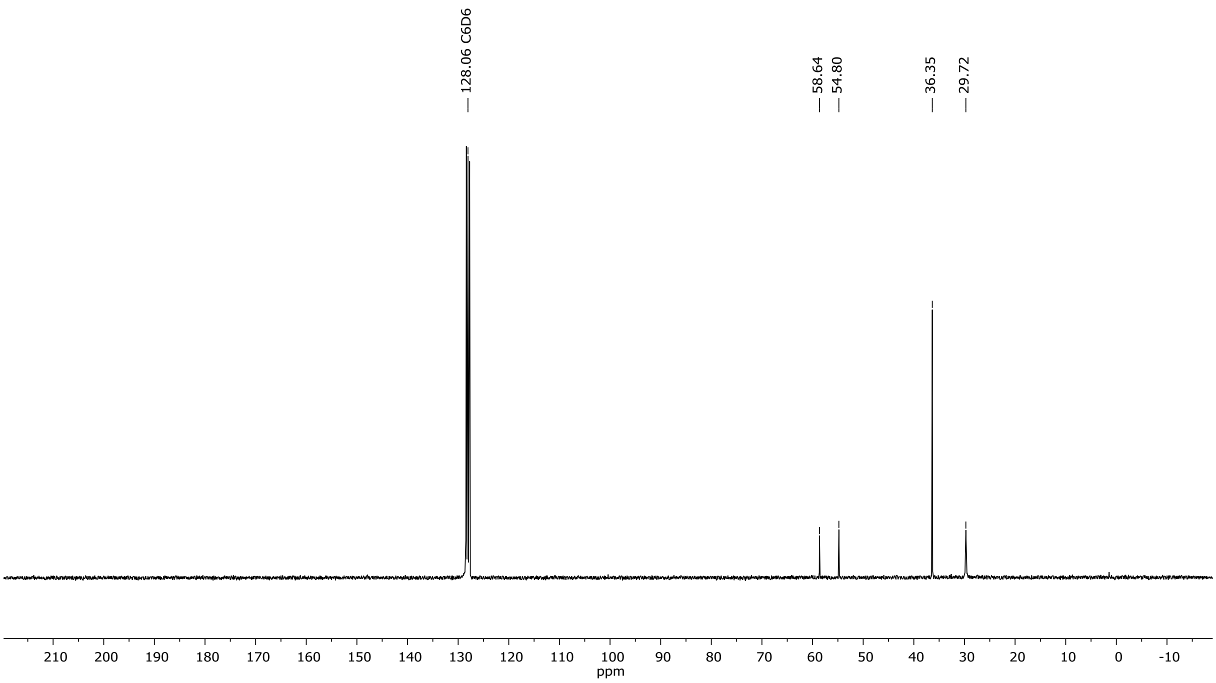

Figure S29. ^{13}C NMR spectrum of **4** (75.5 MHz, C_6D_6, 300K).

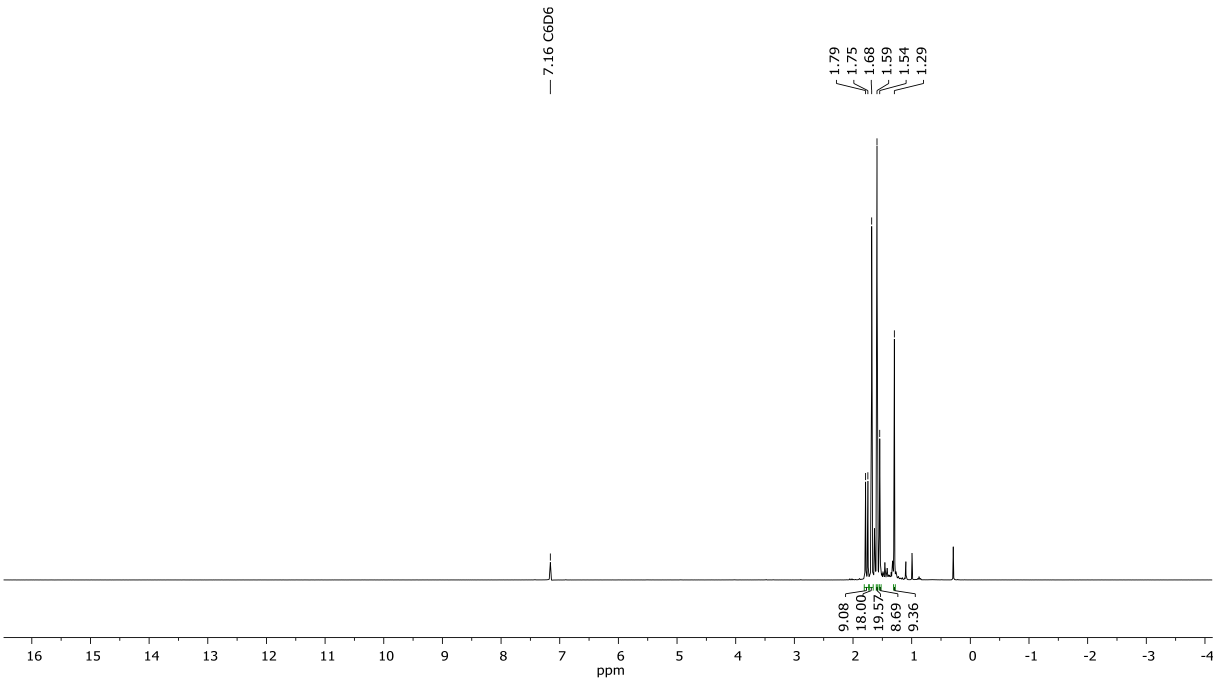

Figure S30. ^{1}H NMR spectrum of **6** (300 MHz, C_6D_6, 300K).

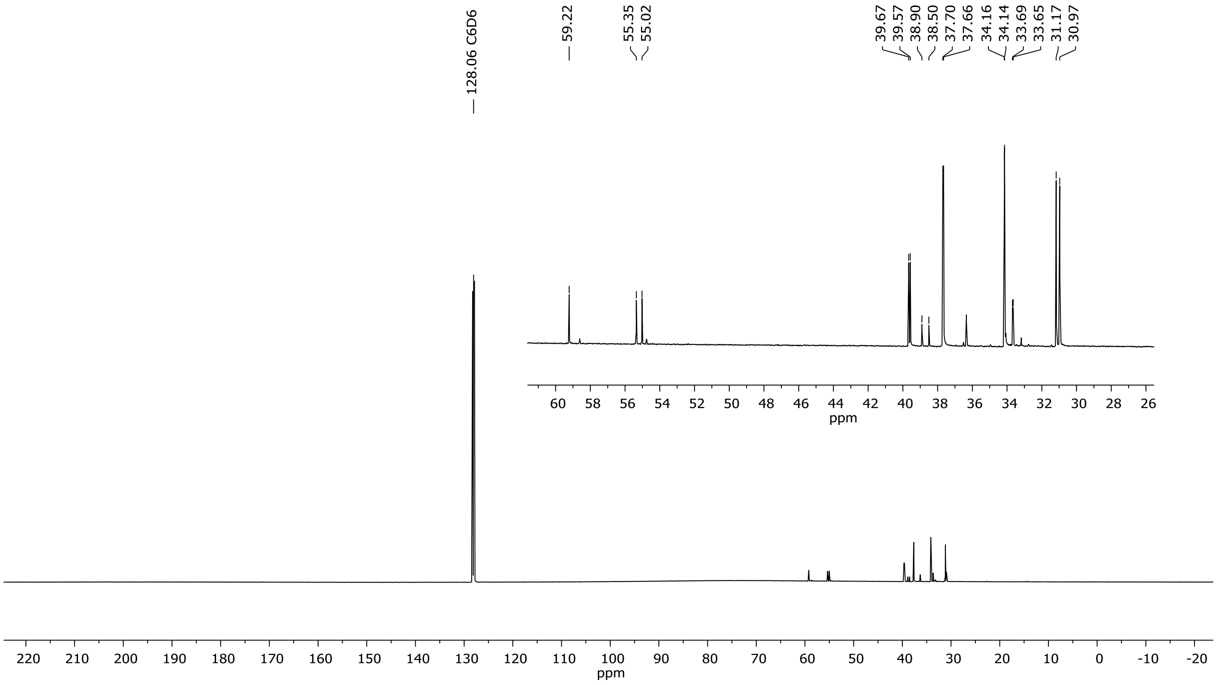

Figure S31. ^{13}C NMR spectrum of **6** (75.5 MHz, C_6D_6, 300K).

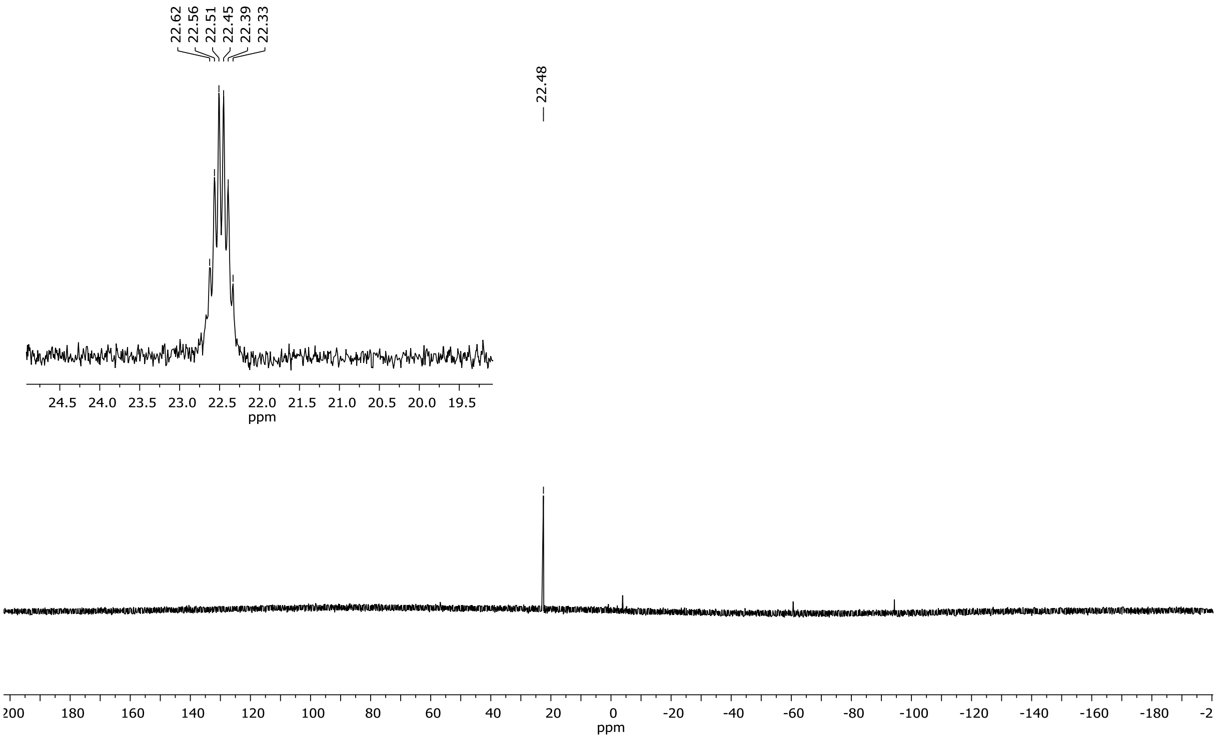

Figure S32. $^{31}P\{^1H\}$ NMR spectrum and ^{31}P NMR spectrum (expansion) of **6** (101 MHz, C_6D_6, 300K).

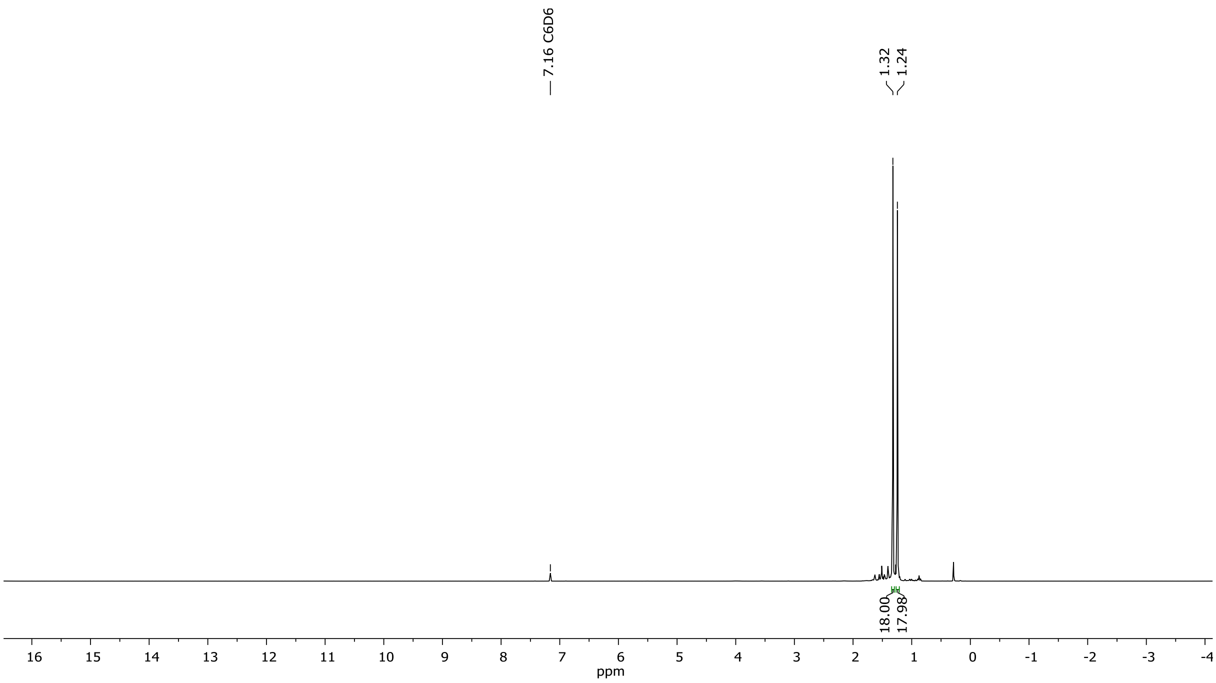

Figure S33. ^{1}H NMR spectrum of **7** (300 MHz, C_6D_6, 300K).

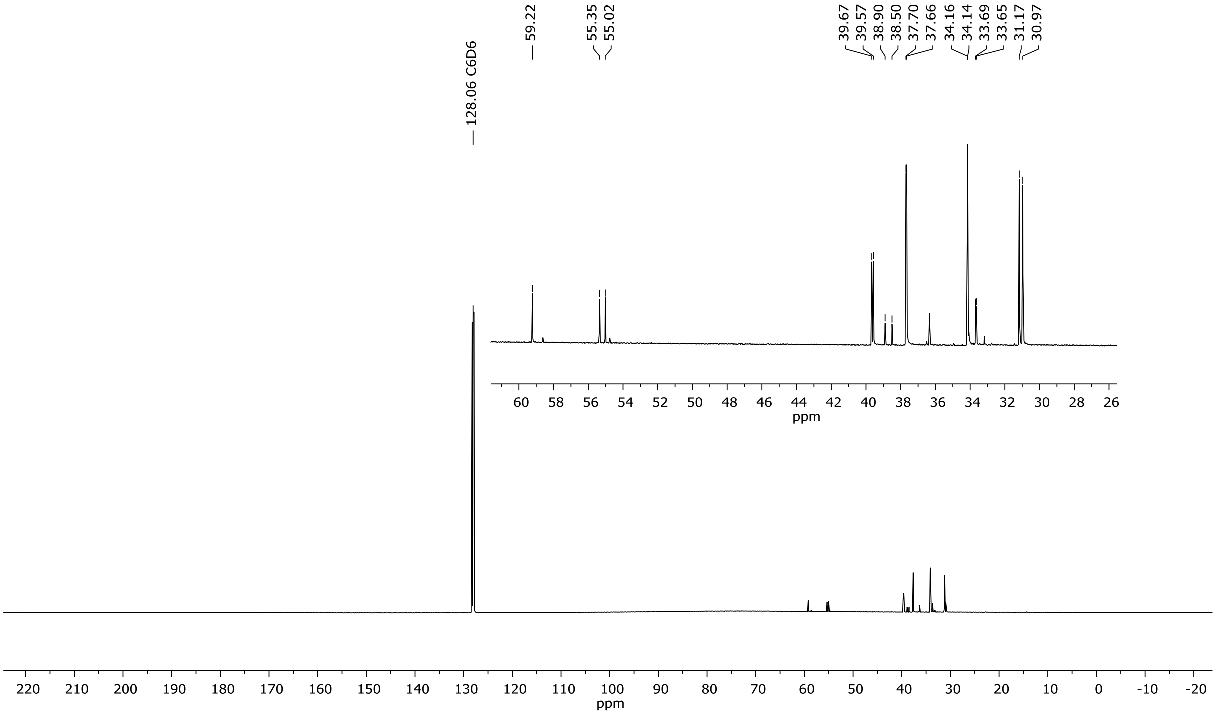

Figure S34. ^{13}C NMR spectrum of **7** (75.5 MHz, C_6D_6, 300K).

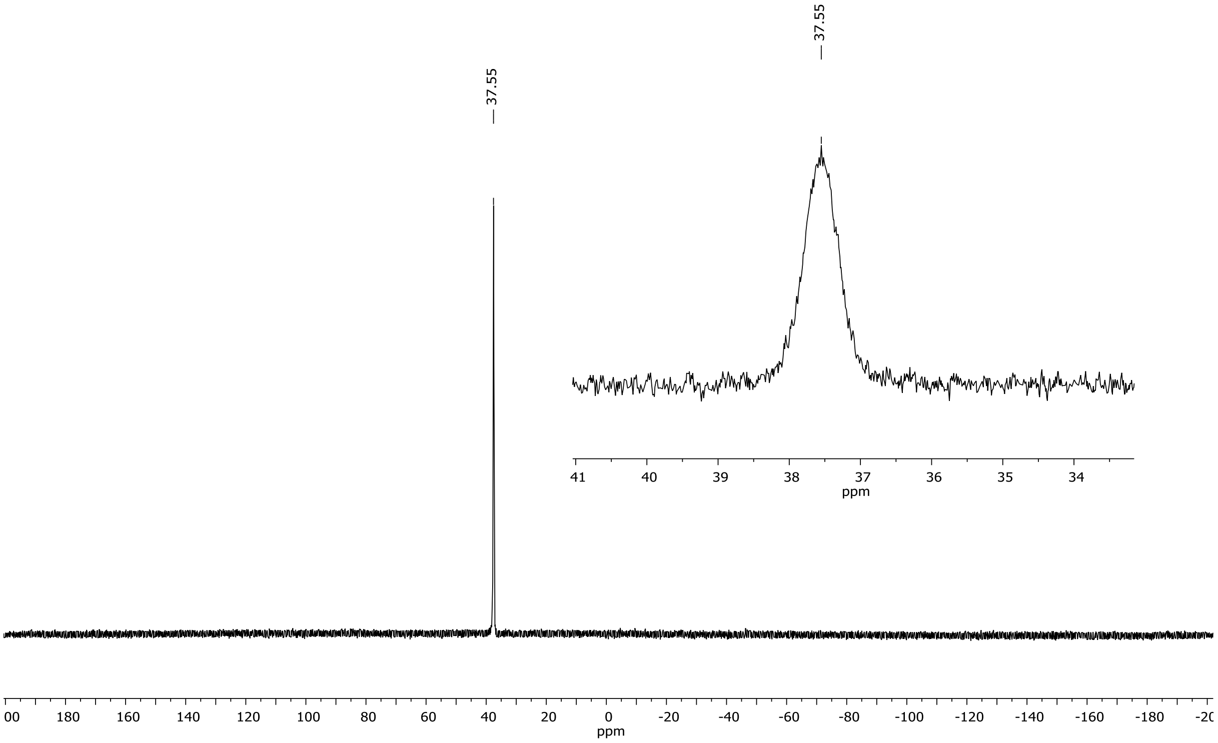

Figure S35. $^{31}P\{^{1}H\}$ NMR spectrum and ^{31}P NMR spectrum (expansion) of **7** (101 MHz, C_6D_6, 300K).

7 References

[1] W. L. F. Armarego, C. L. L. Chai, in *Purif. Lab. Chem.*, Elsevier, **2003**, pp. 80–388.

[2] B. Ringler, M. Müller, C. von Hänisch, *Eur. J. Inorg. Chem.* **2018**, 640–646.

[3] H.-J. Vetter, H. Nöth, W. Jahn, *Z. Anorg. Allg. Chem.* **1964**, *328*, 144–153.

[4] M. Veith, A. Rammo, M. Hans, *Phosphorus. Sulfur. Silicon Relat. Elem.* **1994**, *93*, 197–200.

[5] G. M. Sheldrick, *Acta Cryst. C.* **2015**, *71*, 3–8.

[6] G. M. Sheldrick, *Acta Cryst. A.* **2015**, *71*, 3–8.

[7] O. V. Dolomanov, L. J. Bourhis, R. J. Gildea, J. A. K. Howard, H. Puschmann, *J. Appl. Crystallogr.* **2009**, *42*, 339–341.
